# Effects of Telerehabilitation With Exercise as the Core Component on Peak Oxygen Uptake and Blood Pressure in Patients With Cardiovascular Disease: Systematic Review and Meta-Analysis

**DOI:** 10.2196/95923

**Published:** 2026-09-21

**Authors:** Zhicheng Zhu, Yong Fan, Zhiming Tang, Nangen Song, Youjia Mao, Zijian Zhu

**Affiliations:** 1 Institute of Physical Education, Xinyu University, No. 2666 Sunshine Road, Xinyu, Jiangxi, 338004, China, 86 15679002091; 2 Sports College, Guangxi College of Sports Education, Nanning, Guangxi, China; 3 Chengdu Sport University, Chengdu, Sichuan, China

**Keywords:** telerehabilitation, mHealth, physical activity, cardiovascular disease, meta-analysis, PRISMA, mobile phone, Preferred Reporting Items for Systematic Reviews and Meta-Analyses

## Abstract

**Background:**

Cardiovascular disease (CVD) remains the leading global cause of mortality, and exercise-based cardiac rehabilitation improves cardiorespiratory fitness and reduces recurrent events. However, center-based rehabilitation is constrained. Telerehabilitation has emerged as a scalable alternative, yet prior systematic reviews have generally bundled exercise training with coequal lifestyle components such as health education, dietary counseling, behavior-change techniques, or psychological support, making it difficult to isolate the cardiometabolic contribution of exercise itself.

**Objective:**

This systematic review and meta-analysis quantified the effects of exercise-based telerehabilitation, with exercise as the core therapeutic component, on peak oxygen uptake (VO₂ peak), systolic blood pressure, and diastolic blood pressure in adults with CVD, and examined 5 digital-health dimensions as potential moderators.

**Methods:**

Following PRISMA 2020 (Preferred Reporting Items for Systematic Reviews and Meta-Analyses-2020) and PRISMA-S (Preferred Reporting Items for Systematic Reviews and Meta-Analyses literature search extension) guidelines, PubMed, Cochrane Library, Web of Science, Embase, and MEDLINE were searched from inception to March 20, 2026, supplemented by trial registry searches and forward and backward citation searching. Randomized controlled trials comparing exercise-based telerehabilitation with usual care in adults with CVD were eligible. Risk of bias was assessed with the Cochrane RoB 2 tool (Cochrane Risk of Bias Tool version 2). Random-effects meta-analyses used the Hartung-Knapp-Sidik-Jonkman approach: between-study variance (τ²) was estimated by the Sidik-Jonkman method, and CIs were computed with the Knapp-Hartung adjustment. Prespecified meta-regression and subgroup analyses examined 5 digital-health dimensions: telemedicine modality, guidance type, technology platform, intervention duration, and intervention composition. Certainty of evidence was rated using GRADE (Grading of Recommendations, Assessment, Development, and Evaluation).

**Results:**

Thirteen randomized controlled trials (n=958) were included. Exercise-based telerehabilitation significantly improved VO₂ peak (mean difference [MD]=2.58 mL/kg/min, 95% CI 1.16 to 4.00, *t*_9_=4.10, *P*=.003; 95% prediction interval −1.28 to 6.44; *I*²=74.45%). The prediction interval crossed 0, indicating that the average effect may not be reproduced in every clinical setting. No significant pooled effect was observed for systolic blood pressure (mean difference −1.80 mm Hg, 95% CI −7.42 to 3.81, *P*=.42) or diastolic blood pressure (mean difference −2.00 mm Hg, 95% CI −4.76 to 0.75, *P*=.11). Meta-regression and subgroup analyses did not identify any moderator as a significant source of heterogeneity (all Omnibus *P*>.05), although smartphone or mHealth (mobile health) delivery and professional-led guidance produced larger and more homogeneous VO₂ peak gains. Possible small-study effects for VO₂ peak were detected (Egger test, *P*=.03). GRADE certainty was very low across all outcomes.

**Conclusions:**

Exercise-based telerehabilitation probably improves cardiorespiratory fitness in adults with CVD but provides no convincing evidence of an antihypertensive effect. Telerehabilitation should be considered a patient-centered alternative for individuals unable to access center-based rehabilitation, rather than a uniformly equivalent substitute. Component-isolated trials and hypertensive cohort studies with standardized digital-health reporting are needed.

## Introduction

According to the World Health Organization (WHO), cardiovascular disease (CVD) remains the leading cause of death worldwide and continues to pose a major global health challenge [1]. In 2022, an estimated 19.8 million deaths were attributable to CVD, accounting for approximately 32% of all deaths worldwide [2]. With ongoing population aging and the persistent prevalence of major risk factors, including elevated systolic blood pressure (SBP), unhealthy diet, and high fasting plasma glucose, the burden of CVD remains substantial [3-5]. It has been projected that by 2050, the global number of individuals living with CVD will reach 1.14 billion, with CVD-related deaths rising to 35.6 million [6]. Advances in acute treatment, including percutaneous coronary intervention, coronary artery bypass grafting, and contemporary pharmacological therapies, have markedly reduced early mortality among patients with myocardial infarction and heart failure [7]. However, long-term functional recovery and secondary prevention still require comprehensive management, and exercise-based cardiac rehabilitation (CR) has been shown to reduce cardiovascular mortality and recurrent events in this population [8]. Moreover, long-term adherence to secondary preventive medications remains suboptimal in patients with CVD [9,10]. Accordingly, optimizing long-term outcomes through standardized rehabilitation has become a major priority in CVD management.

CR is a fundamental component of comprehensive CVD care, encompassing cardiovascular risk factor management, psychosocial support, physical activity counseling, and supervised exercise training [11]. Traditional center-based CR has been shown to improve exercise capacity, quality of life, and clinical outcomes; however, its implementation and uptake remain limited by multiple real-world barriers, including insufficient specialist resources, reimbursement difficulties, time constraints, poor adherence, and challenges in long-term follow-up [8,12,13]. In this context, telerehabilitation-based CR has emerged as a promising alternative or adjunct to conventional models. Supported by smartphones, wearable devices, mobile apps, and remote monitoring platforms, this approach can deliver more accessible, continuous, and individualized exercise management beyond the constraints of time and location [14]. It may also enhance patient engagement and self-management through real-time feedback, exercise reminders, data tracking, and remote supervision, making it particularly relevant for home-based rehabilitation and long-term care [15,16]. Consistent with this trend, the European Association of Preventive Cardiology has identified the availability of cardiac telerehabilitation programs as a quality indicator for center accreditation, recognizing telerehabilitation as either an alternative or a complement to center-based CR [17,18].

The current evidence base, however, remains limited and heterogeneous in ways that obscure rather than illuminate the specific contribution of exercise training. Nine prior systematic reviews have examined telerehabilitation or digital health interventions within CR [19-27], but a close examination reveals a consistent gap. Eight of the 9 reviews bundle exercise with health education, dietary counseling, smoking cessation, medication-adherence prompts, and psychosocial support, making it impossible to isolate the independent contribution of exercise training [20-27]. Only Li et al [19] designate peak oxygen uptake (VO₂ peak) as a primary end point, yet that review ranks technology modalities rather than exercise prescriptions and omits blood pressure entirely. Among the remaining reviews, VO₂ peak is either subsumed within composite “functional capacity” constructs [21,22], reported as a nonsignificant secondary finding [20,23,25], or pooled from only 2 to 8 studies [24,26]. Blood pressure is examined in only 3 of the 9 reviews [20,23,27], typically as a secondary outcome with nonsignificant results—a pattern that may reflect intervention dilution rather than a true absence of effect, as the exercise stimulus delivered to any single hemodynamic outcome may have been attenuated when exercise was one of several coequal lifestyle targets [28,29]. Population coverage is equally fragmented, with reviews variously restricted to coronary artery disease (CAD) [23,24], heart failure [21], or constrained by narrow technology or outcome definitions [19,24,25,27]. Consequently, no prior review has simultaneously applied an exercise-centered eligibility filter, designated VO₂ peak as a primary outcome copooled with SBP and diastolic blood pressure (DBP), and enrolled a broad CVD population—the combination needed to test whether telerehabilitation programs in which exercise is the core therapeutic component produce the cardiometabolic signal that bundled multicomponent programs have largely failed to detect.

This systematic review addresses this gap directly and is differentiated from prior reviews along 4 design axes. First, eligibility is restricted to randomized controlled trials (RCTs) in which exercise training is the core therapeutic component of the telerehabilitation program, rather than one of several coequal lifestyle targets—in contrast to the multicomponent eligibility criteria adopted by Cruz-Cobo et al [20], Ramachandran et al [23], and Yang et al [26], which precluded isolation of the exercise stimulus. Second, VO₂ peak is designated a priori as the primary outcome, with SBP and DBP as prespecified secondary outcomes, addressing the underpowered or composite VO₂ analyses of earlier reviews [22,23,25,26] and the systematic omission of blood pressure in 6 of the 9 prior reviews [19,21,22,24-26]. Third, a broad CVD population is enrolled to maximize external validity, rather than being restricted to a single diagnosis as in the heart-failure-only review by Gao et al [21] or the CHD-only reviews by Ramachandran et al [23] and Shi et al [24]. Fourth, and most importantly for the interpretation of digital-health interventions, heterogeneity is interrogated through prespecified meta-regression and subgroup analyses along 5 digital-health-specific dimensions that have not previously been examined together within an exercise-based telerehabilitation framework—whereas earlier reviews have typically ranked technology modalities without analyzing exercise prescription [19], focused on a single delivery platform such as wearable monitoring devices [22] or mobile apps [25], or examined only 1 or 2 design dimensions in isolation [23,27]. The 5 dimensions are telemedicine modality (synchronous vs asynchronous); guidance type (professional-led, patient self-managed, or technology-system-led—separating clinician-directed programs from autonomous patient execution and from interventions in which an intelligent system, such as AI-based motion recognition, assumes the directive role traditionally held by a human professional); technology delivery platform (smartphone or mHealth app-based, wearable sensor-based, or SMS-, web-, or telephone-based); intervention duration (≤12 wk vs >12 wk); and intervention composition (exercise-only vs multicomponent distinguishing programs in which exercise is delivered alone from those that combine exercise with supplementary elements such as health education, dietary counseling, or psychological support). This stratification is positioned to identify which configurations of exercise-based telerehabilitation, rather than telerehabilitation in the aggregate, drive cardiometabolic improvement.

Accordingly, this systematic review and meta-analysis aims to quantify the effect of exercise-based telerehabilitation interventions, compared with usual care without structured exercise training, on VO₂ peak, SBP, and DBP in adults with CVD, and to identify the digital-health-specific characteristics that modify these effects. We hypothesized that exercise-based telerehabilitation would produce a clinically meaningful improvement in VO₂ peak and significant reductions in SBP and DBP relative to usual care, and that synchronous, professional-led or technology-system-led, longer-duration, and exercise-only configurations would yield the largest cardiometabolic gains.

## Methods

### Study Design

This systematic review and meta-analysis was conducted in strict accordance with the PRISMA (Preferred Reporting Items for Systematic Reviews and Meta-Analyses) guidelines and the Cochrane Handbook for Systematic Reviews of Interventions. This study’s protocol was prospectively registered in the PROSPERO (International Prospective Register of Systematic Reviews: CRD420261342287).

Several modifications to the registered protocol (PROSPERO CRD420261342287) were made during the conduct of the review and are declared here for transparency. With respect to outcomes, quality of life and BMI were originally listed as primary outcomes but were removed from the meta-analysis—quality of life because the included trials used heterogeneous instruments (36-Item Short Form Health Survey; SF-36, MacNew, EQ-5D, disease-specific questionnaires) at incompatible time points that precluded meaningful pooling, and BMI because only one included trial reported an extractable end-of-intervention measurement. SBP and DBP, originally grouped as a single outcome, were redesignated as 2 separate prespecified secondary outcomes, with VO₂ peak elevated to the sole primary outcome. With respect to information sources, Web of Science was added to the 4 databases originally specified, expanding the search to 5 databases. The supplementary search was confined to backward citation searching of relevant reviews; we did not contact original trial authors, search conference proceedings, dissertation databases, or trial registers as originally planned, and the final synthesis was restricted to English-language publications despite the protocol imposing no language restriction. Risk of bias was assessed using Cochrane RoB 2 (Cochrane Risk of Bias Tool version 2) only, rather than both RoB-1 and RoB-2 as originally stated. The originally planned *I*²-thresholded model selection has been replaced by a uniform random-effects model with the Hartung-Knapp-Sidik-Jonkman adjustment. Five digital-health-specific moderators (telemedicine modality, guidance type, technology platform, intervention duration, and intervention composition) were prespecified during the analysis phase, operationalizing the broader category of “technology type and intervention duration” mentioned in the protocol. A full GRADE (Grading of Recommendations Assessment, Development, and Evaluation) assessment was added, although the protocol stated certainty would not be assessed, and reporting now follows PRISMA 2020 (expanded) and PRISMA-S (Preferred Reporting Items for Systematic Reviews and Meta-Analyses literature search extension). Safety and feasibility, originally planned to be assessed, are addressed narratively in the Discussion rather than as formal pooled analyses, owing to inconsistent reporting in the primary trials.

### Ethical Considerations

This systematic review and meta-analysis analyzed previously published aggregate-level data and did not involve primary data collection, recruitment of participants, or any form of intervention. Ethical approval and informed consent were obtained by the original investigators of each included trial as documented in their respective publications. As a secondary analysis of deidentified summary statistics extracted from the published literature, this present review did not require separate ethics committee approval or informed consent.

### Eligibility Criteria

A comprehensive literature search was performed in PubMed, the Cochrane Library, Web of Science, Embase, and MEDLINE using a combination of controlled vocabulary terms and free-text terms. The main search terms included “myocardial ischemia,” “coronary artery disease,” and “telemedicine.” No language restrictions were imposed, and all databases were searched from inception to March 20, 2026. To ensure comprehensive study identification, the reference lists of relevant reviews, systematic reviews, and meta-analyses were also manually searched. All retrieved records were imported into EndNote (Clarivate; version 25) for deduplication and record management.

RCTs evaluating the effects of exercise-based telerehabilitation in adults with CVD or related cardiovascular conditions were eligible for inclusion. Studies were included if (1) participants had CAD, ischemic heart disease, myocardial infarction, post–percutaneous coronary intervention status, heart failure, low-risk coronary heart disease, essential hypertension, or had completed CR; (2) the intervention primarily involved exercise-based telerehabilitation, including web-based or mHealth programs, delivered alone or in combination with text messaging, telephone calls, video calls, emails, smartwatches, or related modalities; (3) the control group received usual care without structured exercise training; (4) VO₂ peak was reported as the primary outcome, with SBP and DBP as secondary outcomes; and (5) the study design was an RCT.

Studies were excluded if (1) they involved healthy individuals or patients without CVD or related cardiovascular conditions; (2) the intervention mainly focused on health education, psychoeducation, or lifestyle counseling rather than exercise training; (3) the control group received structured exercise training or telerehabilitation; (4) outcomes related to VO₂ peak or blood pressure were not reported; or (5) the study was a cohort, case-control, cross-sectional, or nonrandomized controlled study, or was published as a conference abstract, review, or meta-analysis.

For the purposes of synthesis, studies were grouped according to telemedicine modality (synchronous vs asynchronous), guidance type (professional-led, patient self-managed, or technology-system-led), technology delivery platform (smartphone or mHealth app-based, wearable sensor-based, or SMS-, web-, or telephone-based), intervention duration (≤12 wk vs >12 wk), and intervention composition (exercise-only interventions that did not incorporate any additional components vs multicomponent interventions that combined exercise with supplementary elements such as health education, dietary counseling, or psychological support) to enable meaningful subgroup comparisons.

### Information Sources

A comprehensive literature search was conducted across 5 electronic databases: PubMed, the Cochrane Library, Web of Science, Embase, and MEDLINE. All databases were searched from inception to March 20, 2026. No language restrictions were imposed. In addition, the reference lists of relevant reviews, systematic reviews, and meta-analyses were manually screened to identify any additional eligible studies. All retrieved records were imported into EndNote (version 25) for deduplication and record management.

### Search Strategy

The search strategy was developed and reported in accordance with the PRISMA-S extension for systematic review search reporting [30]. A systematic literature search was conducted across 5 electronic databases: PubMed, MEDLINE, Embase, Web of Science, and Cochrane Library. Each database was searched individually on its respective platform; no multidatabase simultaneous searching was performed. The search covered the period from database inception to March 20, 2026. No language, publication date, or study design restrictions were applied at the database search stage. No published search filters were used, and the search strategy was developed de novo without adaptation from previous reviews. Full database-specific search strategies, including all Boolean operators, controlled vocabulary terms (MeSH and Emtree), free-text synonyms, and field modifiers, are provided in Section S1 in Multimedia Appendix 1 [31-43].

To supplement the database search, 2 trial registries were searched: ClinicalTrials.gov and the WHO International Clinical Trials Registry Platform, using equivalent key concepts (coronary heart disease, telerehabilitation or telemedicine, and exercise). In addition, both backward and forward citation searching were performed. Backward citation searching was conducted by manually screening the reference lists of all included studies and relevant systematic reviews. Forward citation searching was performed using Web of Science to identify papers that had cited the included studies.

We did not search gray literature databases, dissertation repositories, or conference proceedings, and we did not contact study authors, experts, or manufacturers to obtain additional or unpublished data. No formal peer review of the search strategy was conducted, and no external information specialists were consulted during search strategy development. No search updates or email alerts were established after the initial search was completed. These limitations of the search methodology are acknowledged in the Discussion section.

All retrieved records were imported into EndNote (version 25) for deduplication, which was performed using the software’s built-in duplicate detection function followed by manual verification.

To quantify the degree of citation overlap between the present review and prior systematic reviews of telerehabilitation-based exercise interventions in CVD, we calculated the corrected covered area (CCA) according to Pieper et al [44], using the formula CCA=(N−r)/(*r*×c−r), where N is the total number of included primary publications across all reviews, r is the number of unique primary publications, and c is the number of reviews. CCA values were interpreted using the established thresholds of 0%‐5% (slight), 6%‐10% (moderate), 11%‐15% (high), and >15% (very high overlap) [44]. The complete citation matrix is provided in Section S2 in Multimedia Appendix 1.

### Selection Process

Study selection was performed independently by 2 reviewers in 2 stages. In the first stage, all retrieved records were imported into EndNote (version 25) for deduplication, after which titles and abstracts were screened against the prespecified eligibility criteria; reasons for exclusion were recorded in detail. In the second stage, the full texts of potentially eligible papers were retrieved and independently assessed for final inclusion. Disagreements at either stage were resolved through discussion with a third reviewer until consensus was reached.

### Data Collection Process

Using a prespecified standardized data extraction form, 2 reviewers independently extracted study characteristics and outcome data from each included report. Extracted data were cross-checked to ensure accuracy and consistency. Disagreements were resolved through discussion with a third reviewer until consensus was reached. No automation tools were used, and no additional data were sought from study investigators.

Adverse event reporting and intervention adherence were extracted as secondary safety and feasibility outcomes. For adverse events, we recorded whether each trial included a dedicated adverse event section, the nature and severity of any reported events, and whether events were adjudicated as intervention-related. For adherence, we recorded the metric used, the numerical value reported, and the method of measurement (eg, device-based monitoring, patient logbook, and platform log-in records). Given the anticipated heterogeneity in how these outcomes were operationalized across trials, no attempt was made to pool them quantitatively; they are presented descriptively alongside study characteristics.

### Data Items

The primary outcome was VO₂ peak. Secondary outcomes included SBP and DBP. For each outcome, the means and SDs of both the intervention and control groups at the end of the intervention period were extracted. Where multiple time points were reported, the end-of-intervention measurement was prioritized.

The following study-level and participant-level variables were also extracted: first author, publication year, sample size, mean participant age, intervention type, intervention duration, and proportion of male participants. Where data were missing or unclear, the original report was reexamined. Studies for which the required information remained unavailable after reexamination were excluded from the analysis.

### Study Risk-of-Bias Assessment

The risk of bias of the included studies was independently assessed by 2 reviewers using the revised Cochrane Risk of Bias tool for randomized trials (RoB 2). Disagreements were resolved through consultation with a third reviewer. The following 5 domains were evaluated: bias arising from the randomization process, bias due to deviations from intended interventions, bias due to missing outcome data, bias in measurement of the outcome, and bias in selection of the reported result. Each domain was judged as having a low risk of bias, some concerns, or a high risk of bias, and an overall risk-of-bias judgment was derived for each study accordingly.

### Effect Measures

For all continuous outcomes (VO₂ peak, SBP, and DBP), mean differences (MDs) with 95% CIs were used as the effect measure.

### Synthesis Methods

Eligibility for each synthesis was determined by tabulating the intervention characteristics and outcome domains of each included study and comparing them against the prespecified groupings defined in the eligibility criteria. Studies reporting the same outcome with comparable intervention and control conditions were pooled in the corresponding meta-analysis.

Where SDs were not directly reported, they were calculated in accordance with the Cochrane Handbook for Systematic Reviews of Interventions using the following approaches: when a 95% CI for the difference in means was available, the SD for each group was calculated by dividing the length of the CI by 3.92 and then multiplying by the square root of the sample size; when a *t* value was available, the SD of the difference in means was calculated by dividing the MD by the *t* value and then multiplying by the square root of the sample size; and when actual *P* values obtained from *t* tests were available, the corresponding *t* value was first obtained from a table of the *t* distribution, after which the same formula was applied. No other data conversions were required.

The results of individual studies and pooled syntheses were presented using forest plots. Study-level characteristics were tabulated to provide an overview of the included evidence.

Statistical analyses were performed in Stata (StataCorp; version 18.0) and R (R Foundation; version 4.5.1) with the *metafor* package (version 4.8.0). Given the anticipated clinical and methodological heterogeneity across included studies—arising from differences in populations, intervention protocols, delivery platforms, and follow-up durations—all meta-analyses were conducted using a random-effects model [45]. Between-study variance (τ²) was estimated using the Sidik-Jonkman method, and CIs for the pooled effect estimates were computed with the Knapp-Hartung adjustment (also known as the Sidik-Jonkman adjustment) to provide more adequate coverage when the number of studies is small [46]. With this adjustment, inferences for the overall effect were based on the Student *t* distribution. This combination of methods is hereafter referred to as the Hartung-Knapp-Sidik-Jonkman approach [46]. Heterogeneity was quantified using the Cochran Q test and the *I*² statistic, with significant heterogeneity defined as *P*<.10 or *I*²>50%. For meta-analyses including 10 or more studies, 95% prediction intervals were calculated to distinguish the average treatment effect from the range of true effects expected across different clinical settings [47]. All forest plots and the funnel plot were generated in R using the *metafor* package, with the Knapp-Hartung label explicitly displayed on each forest plot to ensure consistent and transparent presentation across figures. All included studies used nonoverlapping independent subject samples; where a trial included multiple study groups, data from only 1 group were extracted.

For results with significant heterogeneity and sufficient research support (N≥10), we performed meta-regression analysis to explore potential sources of heterogeneity. Prespecified covariates included telemedicine modality (synchronous vs asynchronous), guidance type (professional-led, patient self-managed, or technology-system-led), technology delivery platform (smartphone or mHealth-based, wearable sensor-based, or SMS-, web-, or telephone-based), intervention duration (≤12 wk vs >12 wk), and intervention composition (exercise-only vs multicomponent). Subgroup analyses were conducted along these same dimensions to compare effects across categories. For the guidance type subgroup analysis, professional-led interventions were defined as those in which a qualified health care professional—such as a nurse, coach, or physician—actively directed the intervention through regular structured contact (eg, telephone calls, messaging platforms, or videoconferencing); patient self-managed interventions were defined as those in which participants independently executed the exercise program with asynchronous or automated feedback and without regular proactive contact from a health care provider; and technology-system-led interventions were defined as those in which an intelligent system or algorithm, rather than a human professional, served as the primary agent of real-time guidance, monitoring, or corrective feedback (eg, AI-based motion recognition or automated anomaly detection systems). For the intervention composition subgroup analysis, exercise-only interventions were defined as those in which exercise training was the sole content of the program without any additional components, whereas multicomponent interventions were defined as those that delivered exercise training as the core component alongside supplementary elements such as health education, dietary counseling, or psychological support.

Leave-one-out sensitivity analyses were performed to evaluate the influence of individual studies on the pooled estimates and to assess the robustness of the results.

### Reporting Bias Assessment

When at least 10 studies were available for a given outcome, potential small-study effects were assessed statistically using the Egger regression test.

### Certainty Assessment

The certainty of the body of evidence for each outcome was assessed using the GRADE approach. Evidence was rated as high, moderate, low, or very low based on 5 domains: risk of bias, inconsistency, indirectness, imprecision, and publication bias.

## Results

### Study Selection

A total of 1301 records were retrieved from the databases (PubMed n=210, MEDLINE n=510, Embase n=205, Web of Science n=199, and Cochrane Library n=177). In addition, 136 records were identified through other methods, including 132 from trial registries (ClinicalTrials.gov n=129 and WHO International Clinical Trials Registry Platform n=3) and 4 from citation searching (backward n=3 and forward n=1).

For the database searches, 436 duplicate records were removed using EndNote (version 25), leaving 865 records for screening. Titles and abstracts were screened against the eligibility criteria, and 808 records were excluded. Subsequently, 57 reports were sought for full-text retrieval, of which 1 could not be retrieved, leaving 56 reports for full-text eligibility assessment. Of these, 25 were excluded for not meeting the inclusion criteria, 20 were excluded because the interventions evaluated were not exercise-based remote rehabilitation, and 1 was excluded because it used a nonrandomized controlled design, resulting in 10 studies included from database searches.

For the 136 records identified through other methods, all underwent screening. Of these, 42 records were duplicates with database search results, 34 records were ongoing trials without published results, and 56 records did not meet the inclusion criteria, leaving 4 reports for full-text eligibility assessment. After full-text assessment, 1 was excluded for not matching the inclusion criteria, resulting in 3 studies included from this pathway.

**Figure 1.**
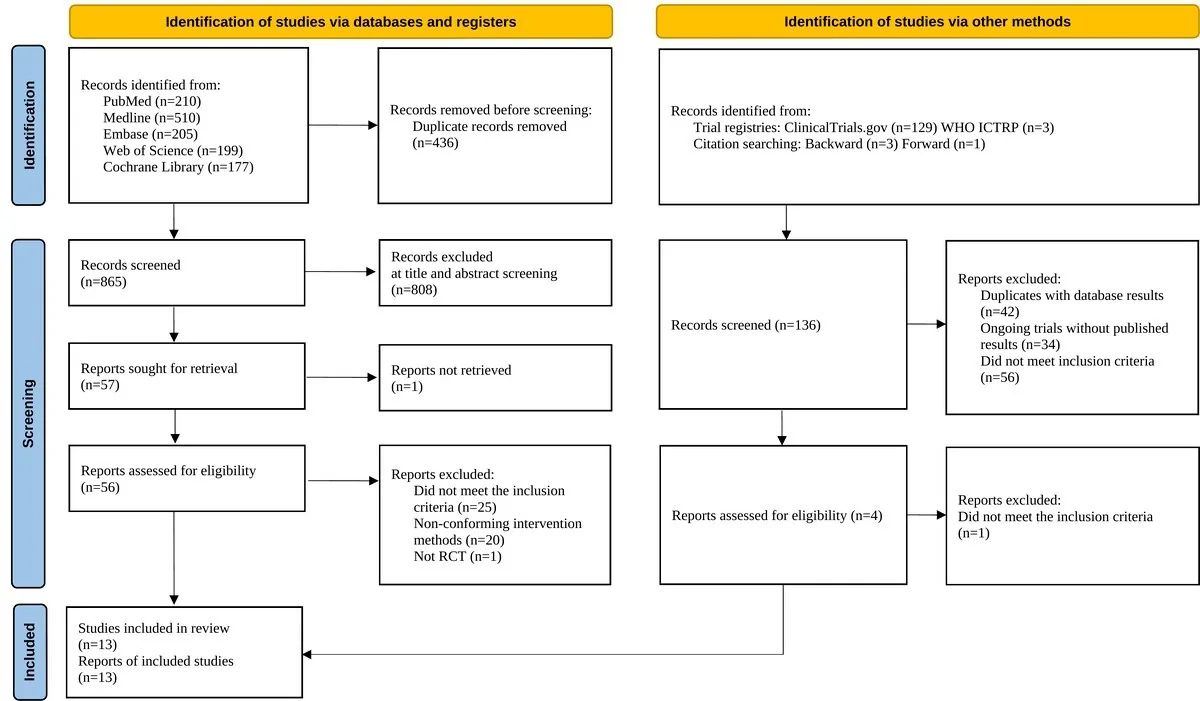
PRISMA 2020 flow diagram of the study selection process, showing records identified from the 5 databases and from other sources, duplicate records removed before screening, records screened and excluded, reports assessed for eligibility with reasons for exclusion, and the 13 studies included in the review. PRISMA: Preferred Reporting Items for Systematic Reviews and Meta-Analyses; RCT: randomized controlled trial.

Finally, a total of 13 studies [31-43] were included in the meta-analysis, comprising 10 studies from database searches and 3 studies from other methods. The study screening process is shown in Figure 1.

Among the 47 papers excluded during the full-text evaluation stage, the main reasons for exclusion were (1) not meeting the inclusion criteria (n=26, including 25 from database searches and 1 from other methods), for example, the outcome indicators of the study by Leemrijse et al [48] did not meet the criteria; (2) the study design was a non-RCT (n=1); (3) although the interventions included remote methods, the core content was not exercise-based remote rehabilitation (n=20).

To quantify the degree of citation overlap between the present review and previous systematic reviews on telerehabilitation-based exercise interventions in CVD, we calculated the CCA following Pieper et al [44], excluding the umbrella review by Shi et al [24], which does not independently search for primary trials. The CCA was 6.6% (N=19, *r*=13, c=8), falling within the 6%‐10% range conventionally interpreted as moderate overlap [10]. This indicates that, despite addressing a related clinical question, the present review draws on a largely distinct primary-trial evidence base relative to prior reviews, consistent with our exercise-isolated eligibility criteria and broad cardiovascular-disease enrollment. The full citation matrix is provided in Section S2 in Multimedia Appendix 1.

### Study Characteristics

Thirteen studies published in English were included, comprising a total of 958 participants. The studies were published between 2007 and 2026 and were conducted in China [35,39-41,43], Iran [32], Belgium [31,34], the United States [33], New Zealand [36], Israel [37], Brazil [38], and Canada [42]. All studies reported participant age and sex distribution. Mean age ranged from 11.2 to 69.0 years, the proportion of male participants ranged from 47.5% (19/40) to 90.77% (59/65), and sample sizes ranged from 7 to 86.

In the studies reporting VO₂ peak, in terms of telemedicine modality, 5 studies [31,33,34,36,38] used asynchronous delivery, while the remaining 5 studies [37,39-41,43] used synchronous communication. Regarding guidance type, 5 studies [31,33,38,40,43] were classified as professional-led, involving active and structured contact with nurses, coaches, or physicians via telephone, messaging platforms, or video calls; 3 studies [34,36,37] were classified as patient self-managed, in which participants independently executed the exercise program with asynchronous or automated feedback and without regular proactive contact from a health care provider; and 2 studies [39,41] were classified as technology-system-led, in which an intelligent system served as the primary agent of real-time guidance through AI-based motion recognition or automated anomaly detection. With respect to technology delivery platform, 5 studies [33,39-41,43] used smartphone or mHealth app-based platforms, 2 studies [31,34] used wearable sensor-based systems, and 3 studies [36-38] used SMS-, web-, or telephone-based platforms. Concerning intervention duration, 6 studies [31,33,38,40,41,43] had durations of ≤12 weeks, whereas 4 studies [34,36,37,39] exceeded 12 weeks. Regarding intervention composition, 8 studies [31,33,34,36,37,40,41,43] were multicomponent interventions that combined exercise training with supplementary elements such as health education, dietary counseling, or psychological support, while 2 studies [38,39] consisted of exercise-only interventions without any additional components. Detailed study characteristics are presented in Table 1.

**Table 1.** Characteristics of the 13 randomized controlled trials included in this systematic review and meta-analysis^a^.

Study country	Population^b^ (P)	Intervention^c^ (I)	Control group intervention measures and duration	Outcome^c^ (O)^d^
Avila et al [31]; Belgium	CAD^e^ 30 vs 30 58.6 (13) vs 61.7 (7.7) 86.7% vs 90%	After completing a 3-month ambulatory CR^f^ program Home-based exercise with telemonitoring guidance Personalized aerobic prescription + Garmin 12 weeks At least 150 min per week	Conventional care 12 weeks	S^g^: (1) P^h^: (2); (3); (4)
Li et al [43]; China	CHD^i^ 34 vs 34 11.2 (2.7) vs 11.2 (2.6) 55.9% vs 55.9%	After baseline cardiopulmonary exercise testing and risk stratification Home-based mHealth^j^ CR via WeChat (telehealth) Individualized exercise prescription + nurse-led remote monitoring + health education (3 times/wk) + behavior change techniques (goal setting, self-monitoring, problem solving) + basic exercise equipment 12 weeks 4 sessions/wk, 45‐60 min/session	Conventional care 12 weeks	S: (1)
Dehghani et al [32]; Iran	PCI^k^ 40 vs 40 49.77 (7.88) vs 51.45 (7.46) 47.5% vs 50%	At least 2 months after PCI Home-based exercise with telemonitoring guidance Supervised exercise training 8 weeks 3 sessions per week (total of 40 sessions). Walking: 30 min/day, with step count progressively increasing 15% per week	Conventional care 8 weeks	P: (3); (4)
Duscha et al [33]; United States	Patients graduating from CR 16 vs 9 59.9 (8.1) vs 66.5 (7.2) 81.2% vs 66.7%	After completing 36 on-site CR sessions mHealth program using physical activity trackers and health coaching Fitbit activity tracker 12 weeks Daily step goals; coaching calls 1‐2 times per week	Conventional care 12 weeks	S: (1)
Frederix et al [34]; Belgium	CAD 40 vs 40 58 (9) vs 63 (10) 81% vs 85%	After week six of their conventional phase II CR Physical activity telemonitoring program Motion sensor (3D accelerometer) worn continuously 18 weeks Continuous monitoring, weekly feedback.	Conventional care 18 weeks	S: (1)
Fang et al [35]; China	PCI 40 vs 40 60.24 (9.35) vs 61.41 (10.16) 63.6% vs 61.8%	After being discharged Home-based cardiac telerehabilitation Outdoor walking or jogging with real-time physiological monitoring (sensor, smartphone app) 6 weeks Outdoor walking or jogging no less than 3 times/wk	Conventional care 6 weeks	P: (3); (4)
Maddison et al [36]; New Zealand	IHD^l^ 85 vs 86 61.4 (8.9) vs 69.0 (9.5) 81% vs 81%	Clinically stable outpatients Mobile phone and internet-based intervention (HEART^m^ program) Personalized automatic SMS package (118 in 24 wk) and a secure website containing video information 24 weeks Text messages: 6/wk (first 12 wk), 5/wk (next 6 wk), 4/wk (last 6 wk)	Conventional care 24 weeks	S: (1)
Nabutovsky et al [37]; Israel	Myocardial infarction, coronary intervention or heart failure 45 vs 24 56.6 (12.3) vs 54.5 (12.2) 80% vs 83.3%	After being referred to the outpatient CR institute and declining CBCR^n^ Asynchronous home-based CR (HBCR^o^) Smart watch detection + Datos Health 6 months Encouraged to engage in PA^p^ according to goals	Conventional care 6 months	S: (1)
Salvetti et al [38]; Brazil	Patients with low-risk coronary heart disease 19 vs 20 53 (8) vs 54 (9) 74% vs 75%	After a coronary event (phase III CR) Home-based training program Individualized training based on target heart rate (60%‐80% peak h), exercise log, biweekly telephone monitoring by a doctor 3 months Walking 3 times per week for 30 minutes on nonconsecutive days	Conventional care 3 months	S: (1) P: (3); (4)
Song et al [39]; China	Coronary heart disease 48 vs 48 54.17 (8.76) vs 54.83 (9.13) 89.6% vs 83.33%	After discharge and enrollment Smartphone-based telemonitored exercise rehabilitation Smartphone software; heart rate band; exercise prescription; by 6 months Exercise 3‐5 times/wk, 30 min/session. Feedback once a week	Conventional care 6 months	S: (1)
Wan et al [40]; China	PCI 65 vs 65 62.32 (9.63) vs 61.35 (9.18) 90.77% vs 81.54%	At discharge from hospital after PCI WeChat-based brisk walking program Rehabilitation guidance; WeChat group; exercise prescription 12 weeks 3 d/wk for first 4 weeks, then 5 d/wk for next 8 weeks; 30 min/session	Conventional care 12 weeks	S: (1)
Yao et al [41]; China	Patients with essential hypertension 31 vs 31 50.48 (9.44) vs 55.42 (12.86) 61.3% vs 54.8%	After enrollment AI recognition-based telerehabilitation Applications; online consultation; personalized exercise prescription 8 weeks Prescription pushed 5 times/wk, required to complete at least 3 times/wk; 30‐50 min/session	Conventional care 8 weeks	S: (1) P: (3); (4)
Zutz et al [42]; Canada	CVD^q^ patients without prior rehabilitation 8 vs 7 58 (4) vs 59 (12) 87.5% vs 57%	While on the waiting list for a local hospital-based CRP^r^ Internet-based vCRP^s^ Sports session; heart rate monitoring 12 weeks Nonconstant	Conventional care 12 weeks	P: (2)

a Characteristics of the 13 randomized controlled trials included in this systematic review and meta-analysis of exercise-based telerehabilitation vs usual care in adults with cardiovascular disease, including coronary artery disease, post–percutaneous coronary intervention, ischemic heart disease, heart failure, congenital heart disease, low-risk coronary heart disease, and essential hypertension. Studies were conducted between 2007 and 2026 in China, Iran, Belgium, the United States, New Zealand, Israel, Brazil, and Canada. The table reports first author and country, population characteristics (medical diagnosis, sample size by group, age, and sex), intervention details (timing of initiation, telehealth modality, components, duration, and frequency), control group description and duration, and reported outcomes (VO₂ peak, SBP, and DBP) classified as primary or secondary.

b (1) Medical diagnosis; (2) sample (intervention group vs control group); (3) age (mean, SD; intervention group vs control group); (4) gender (%, male, intervention group vs control group).

c (1) Time of start; (2) types of telehealth cardiac rehabilitation (d or times/wk).

d (1) Peak oxygen uptake; (2) BMI; (3) systolic blood pressure; (4) diastolic blood pressure.

e CAD: coronary artery disease.

f CR: cardiac rehabilitation.

g S: secondary end point.

h P: primary end point.

i CHD: congenital heart disease.

j mHealth: mobile health.

k PCI: post–percutaneous coronary intervention.

l IHD: ischemic heart disease.

m HEART: heart exercise and remote technologies.

n CBCR: center-based cardiac rehabilitation.

o HBCR: home-based cardiac rehabilitation.

p PA: physical activity.

q CVD: cardiovascular disease.

r CRP: cardiac rehabilitation program.

s vCRP: “virtual” cardiac rehabilitation program.

Adverse event reporting and intervention adherence varied considerably across included trials. Regarding safety, 10 of the 13 trials included an explicit adverse event statement, of which 9 trials declared that no serious exercise-related adverse events occurred during the intervention period [31,32,37-43]. One trial [36] reported 31 serious adverse events across 22 participants, of which only 1 participant, a hospitalization following a cycling accident, was adjudicated as intervention-related. The remaining 3 trials [33-35] did not include a dedicated adverse event section [34]; instead reported rehospitalization rates as a clinical end point rather than as formally adjudicated safety data. A per-study summary of adverse event reporting is provided in Section S3 in Multimedia Appendix 1.

Adherence to the prescribed intervention was quantified using heterogeneous metrics across trials. Exercise sessions per week were reported by 4 trials [31,37-39], daily step counts by 2 trials [33,37], platform login frequency by 1 trial [42], and message or video engagement rates by 1 trial [36]. One trial reported the percentage of prescribed sessions completed [43], and 1 trial applied an attendance threshold as an eligibility criterion rather than reporting adherence as an outcome [32]. One trial described adherence as “good” without supplying numerical data [35], and 3 trials provided no adherence data whatsoever [34,40,41].

### Risk of Bias in Studies

**Figure 2.**
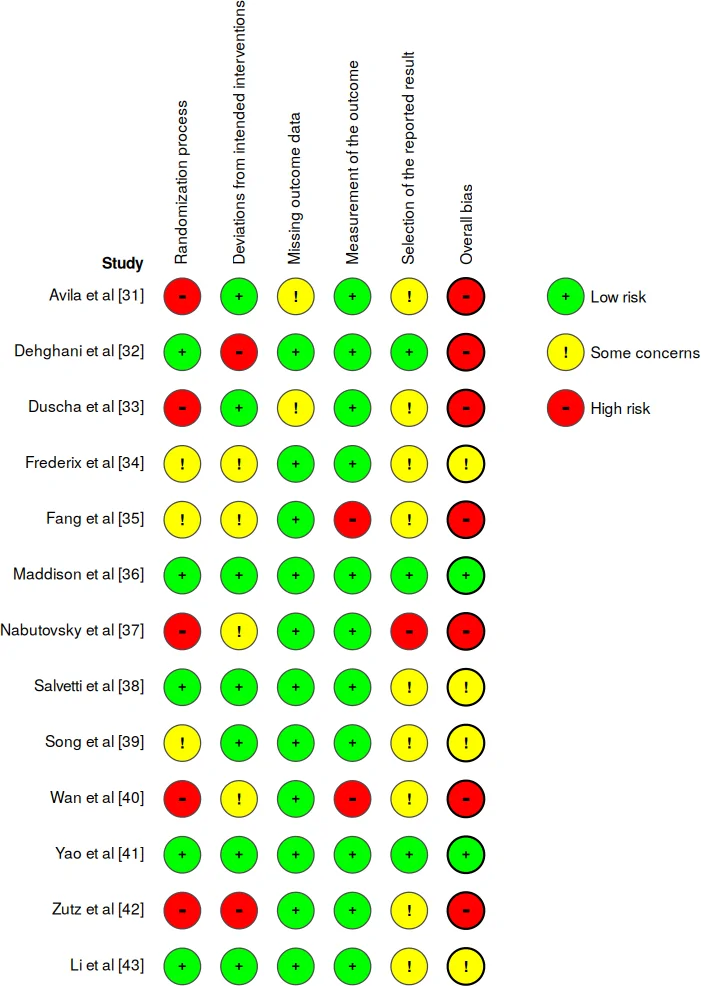
Risk of bias assessment of each of the 13 included randomized controlled trials of exercise-based remote rehabilitation using the Cochrane Risk of Bias tool version 2 (RoB 2) across 5 domains: randomization process, deviations from intended interventions, missing outcome data, outcome measurement, and selection of reported results [31-43].

**Figure 3.**
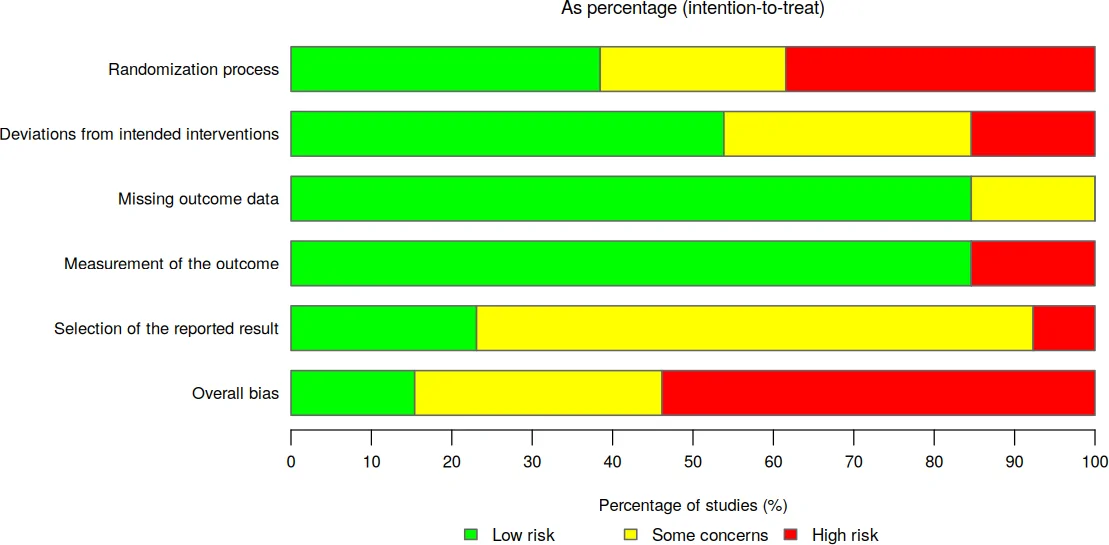
Summary of Cochrane Risk of Bias tool version 2 (RoB 2) judgments across 5 domains (randomization process, deviations from intended interventions, missing outcome data, outcome measurement, and selection of reported results) and overall bias ratings for 13 included randomized controlled trials of exercise-based remote rehabilitation. Overall, 15.4% (2/13) of studies were rated low risk, 30.8% (4/13) had some concerns, and 53.8% (7/13) were rated high risk of bias.

The RoB 2 was used to assess the methodological quality of all 13 included studies. The assessment covered 5 dimensions: randomization process, deviation from expected intervention, missing outcome data, outcome measurement, and selectivity in reporting results, and a comprehensive assessment of the overall risk of bias was given. Detailed assessment results for each study are shown in Figure 2 [31-43], and the summarized percentage results are shown in Figure 3.

Regarding the randomization process, 38.5% (5/13) of the studies were assessed as having low risk of bias, 23.1% (3/13) had some concerns, and 38.5% (5/13) were assessed as having high risk of bias. The high risk of bias was mainly due to inadequate description of the random sequence generation methods or failure to report allocation concealment schemes, making it one of the most prominent dimensions in this assessment.

Regarding deviations from intended interventions, 53.8% (7/13) of the studies were assessed as having low risk of bias, 30.8% (4/13) as having some concern, and 15.4% (2/13) as having high risk of bias. Due to the nature of exercise-based telerehabilitation, participant blinding was not feasible in all studies, and some studies had shortcomings in handling intervention adherence and intention-to-treat analyses.

Regarding missing outcome data, 84.6% (11/13) of the studies were assessed as having low risk of bias, 15.4% (2/13) as having some concern, and no studies were assessed as having high risk of bias, suggesting that the overall handling of outcome data completeness was relatively appropriate across studies.

Regarding measurement of the outcome, 84.6% (11/13) of the studies were assessed as having low risk of bias, 15.4% (2/13) as having high risk of bias, and no studies had some concern. The widespread use of objective measurements (eg, VO₂ peak and blood pressure) helps reduce the risk of measurement bias.

Regarding selection of the reported result, 23.1% (3/13) of the studies were rated as low risk of bias, 69.2% (9/13) had some concerns, and 7.7% (1/13) were rated as high risk of bias. This dimension represents the area with the highest concentration of bias concerns, as most studies did not undergo preregistration or predefine the primary outcome, making it difficult to rule out the risk of selective reporting.

Regarding overall bias, based on the combined assessment of the 5 dimensions, 15.4% (2/13) of the studies were classified as having low overall risk of bias, 30.8% (4/13) had some concerns, and 53.8% (7/13) were classified as having high overall risk of bias. The overall bias level is primarily driven by 2 dimensions: inadequate randomization and selective reporting of results. These methodological limitations should be carefully considered when interpreting the results of this meta-analysis.

### Results of Individual Studies

**Figure 4.**
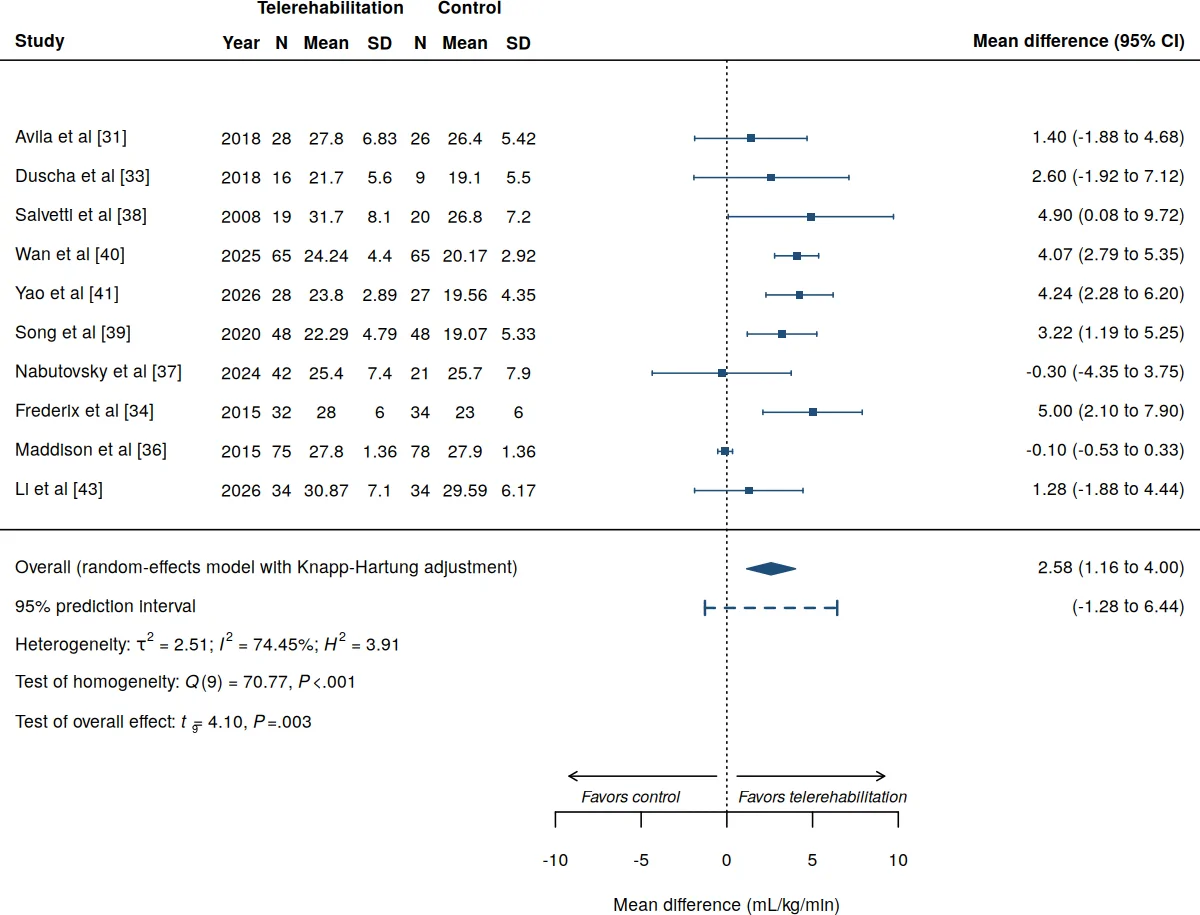
Forest plot of the effects of exercise-based telerehabilitation vs usual care on VO₂ peak (mL/kg/min) in patients with cardiovascular disease, based on 10 randomized controlled trials (n=749) with intervention durations ranging from 8 to 24 weeks. Pooled analysis using a random-effects Hartung-Knapp-Sidik-Jonkman model showed a significant improvement in VO₂ peak (mean difference 2.58 mL/kg/min, 95% CI 1.16 to 4.00, *t*_9_=4.10, *P*=.003), with substantial heterogeneity (*I*²=74.45%) [31,33,34,36-41,43].

**Figure 5.**
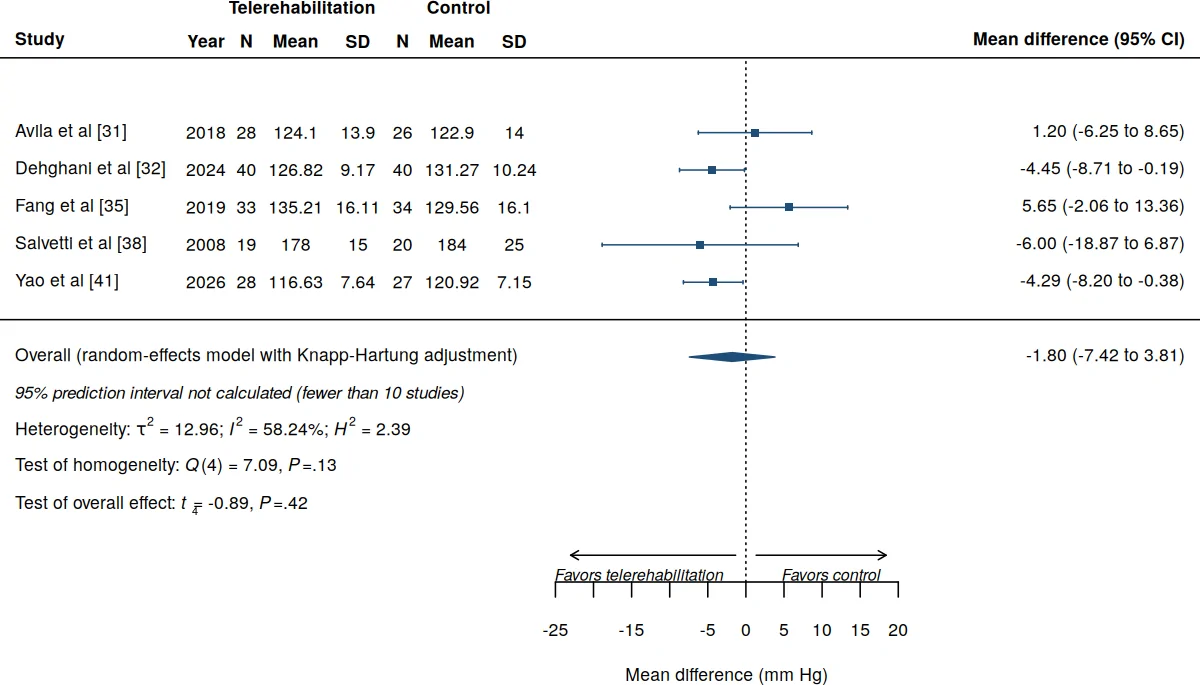
Forest plot of the effects of exercise-based telerehabilitation vs usual care on systolic blood pressure (mm Hg) in patients with cardiovascular disease, based on 5 randomized controlled trials (n=295; intervention: n=148, control: n=147) published between 2008 and 2026. Pooled analysis using a random-effects Hartung-Knapp-Sidik-Jonkman model showed no statistically significant effect on systolic blood pressure (mean difference −1.80 mm Hg, 95% CI −7.42 to 3.81, *t*_4_=−0.89, *P*=.42), with moderate heterogeneity (*I*²=58.24%) [31,32,35,38,41].

**Figure 6.**
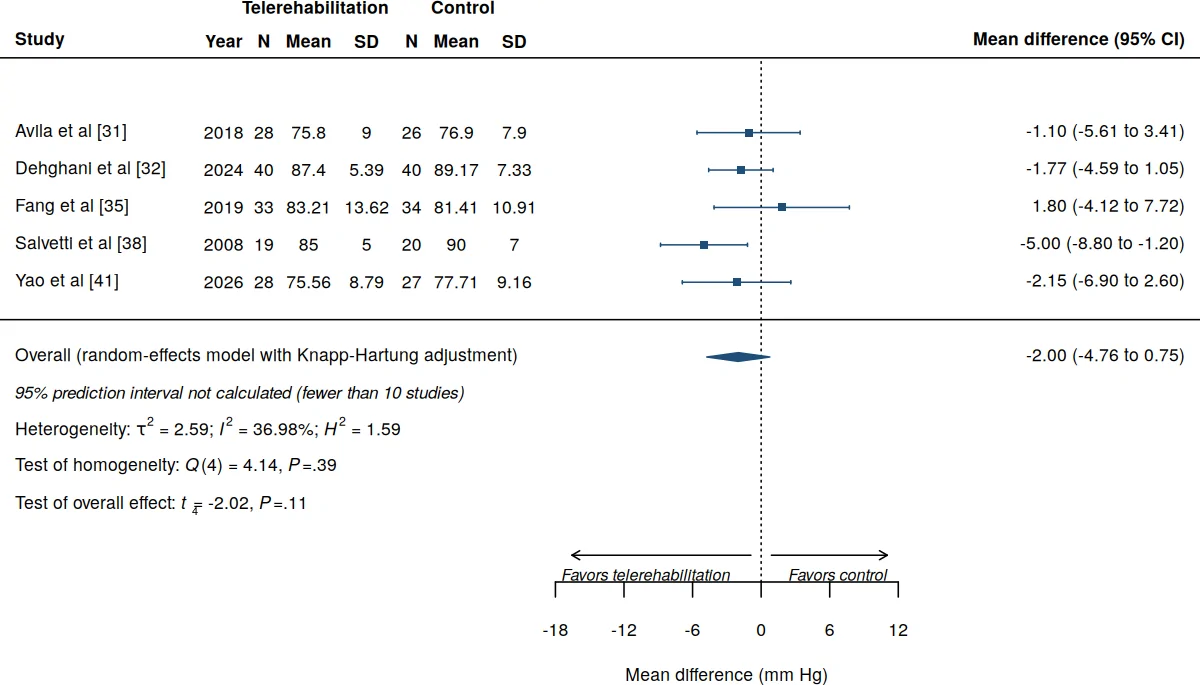
Forest plot of the effects of exercise-based telerehabilitation vs usual care on diastolic blood pressure (mm Hg) in patients with cardiovascular disease, based on 5 randomized controlled trials (n=295; intervention: n=148, control: n=147) published between 2008 and 2026. Pooled analysis using a random-effects Hartung-Knapp-Sidik-Jonkman model showed no statistically significant effect on diastolic blood pressure (mean difference −2.00 mm Hg, 95% CI −4.76 to 0.75, *t*_4_=−2.02, *P*=.11), with low to moderate heterogeneity (*I*²=36.98%) [31,32,35,38,41].

Summary statistics (means and SDs) and effect estimates (MDs with 95% CIs) for each included study are presented in the forest plots (Figures 4-6) [31-41,43]. Individual study data are displayed alongside the pooled effect estimates to facilitate study-level evaluation and comparison.

### Results of Syntheses

#### VO₂ Peak

Ten studies included in the VO₂ peak analysis involved 749 participants from multiple countries, with intervention durations ranging from 8 to 24 weeks. These studies used both assisted and automated remote rehabilitation modalities. According to the RoB 2 assessment, a significant proportion of contributing studies presented a high risk of bias or some concerns, particularly in the randomization process (5/13, 38.5% high risk) and selective reporting of outcomes (9/13, 69.2% concerns). These methodological limitations should be fully considered when interpreting the pooled effect.

A pooled analysis using the Hartung-Knapp-Sidik-Jonkman random-effects model showed that, compared to usual care, exercise-based telerehabilitation significantly improved VO₂ peak in patients with CVD (MD 2.58 mL/kg/min, 95% CI 1.16 to 4.00, *t*_9_=4.10, *P*=.003; Figure 4). However, substantial statistical heterogeneity existed among the studies (τ²=2.51, *I*²=74.45%, *H*²=3.91, Q(9)=70.77, *P*<.001), suggesting substantial differences in the true effect size among the studies. The 95% prediction interval (−1.28 to 6.44) crossed 0, indicating that the intervention’s improvement on VO₂ peak may not be significant in certain study scenarios or clinical contexts. Therefore, the pooled effect estimate should be understood as the average effect across study scenarios, rather than an effect prediction universally applicable to all clinical contexts. The mean, SD, and effect estimate for each included study are detailed in Figure 4.

#### SBP

Five studies included in the SBP analysis involved a total of 295 participants (148 in the intervention group and 147 in the control group), published between 2008 and 2026. According to the RoB 2 assessment, contributing studies have methodological limitations in randomization and selective reporting of results, which should be considered when interpreting pooled effects.

A pooled analysis using the Hartung-Knapp-Sidik-Jonkman random-effects model showed that the effect of exercise-based telerehabilitation on SBP was not statistically significant compared to usual care (MD −1.80 mm Hg, 95% CI −7.42 to 3.81, *t*_4_=−0.89, *P*=.42; Figure 5). Moderate statistical heterogeneity existed among the studies (τ²=12.96, *I*²=58.24%, *H*²=2.39, Q(4)=7.09, *P*=.13). The CIs were wide and crossed 0, indicating significant uncertainty in the direction and magnitude of the effect. The mean, SD, and effect estimates for each group included in the study are detailed in Figure 5.

#### DBP

Five studies included in the DBP analysis involved a total of 295 participants (148 in the intervention group and 147 in the control group), published between 2008 and 2026. According to the RoB 2 assessment, contributing studies have certain methodological limitations in randomization and selective reporting of results, which should be considered when interpreting pooled effects.

A pooled analysis using the Hartung-Knapp-Sidik-Jonkman random-effects model showed that the effect of exercise-based telerehabilitation on DBP was not statistically significant compared to usual care (MD −2.00 mm Hg, 95% CI −4.76 to 0.75, *t*_4_=−2.02, *P*=.11; Figure 6). Interstudy heterogeneity was low to moderate (τ²=2.59, *I*²=36.98%, *H*²=1.59, Q(4)=4.14, *P*=.39), suggesting relatively limited variability in effect sizes among studies. The CIs cross 0, indicating significant uncertainty regarding the direction and magnitude of the effect. See Figure 6 for the mean, SD, and effect estimates for each included study.

### Meta-Regression

For the primary outcome VO₂ peak, a random-effects meta-regression model was used to explore potential sources of heterogeneity, incorporating 5 prespecified covariates: intervention duration, telemedicine mode, guidance type, technology delivery platform, and intervention composition. Detailed results are shown in Table 2.

**Table 2.** Meta-regression analysis of moderators of the effect of exercise-based telerehabilitation on VO₂ peak (mL/kg/min), based on 10 randomized controlled trials (n=749). Each moderator was analyzed in a separate univariate random-effects meta-regression using the HKSJ^a^ approach. None of the moderators reached statistical significance at the 0.05 threshold (all Omnibus *P*>.05).

	Moderator and comparison (vs reference)	ΔMD^b^ (mL/kg/min)	95% CI	*t* test (*df*)*^c^*	*P* value	Omnibus *P*
Intervention duration	>12 wk vs ≤12 wk	−1.38	−4.26 to 1.51	−1.10 (8)	.30	.30
Telemedicine mode	Asynchronous vs synchronous	0.79	−2.23 to 3.81	0.60 (8)	.56	.56
Type of guidance	Patient self-managed vs professional-led Technology-system-led vs professional-led	−1.59 0.84	−4.98 to 1.80 −2.85 to 4.53	−1.13 (7) 0.54 (7)	.30 .61	.36
Technology delivery platform	Smartphone or mHealth^d^ vs wearable SMS, web, or telephone vs wearable	0.98 −1.25	−2.66 to 4.61 −5.63 to 3.12	0.65 (7) −0.66 (7)	.55 .52	.41
Intervention composition	Multicomponent vs exercise-only	−1.38	−5.28 to 2.53	−0.83 (8)	.44	.44

a HKSJ: Hartung-Knapp-Sidik-Jonkman.

b ΔMD: change in pooled mean difference of VO₂ peak (in mL/kg/min) associated with each comparison category, relative to the reference category. Each three-level moderator (type of guidance, technology delivery platform) was entered using dummy coding, yielding 2 pairwise contrasts plus an Omnibus test for the joint moderator effect.

c *t*: test statistic from Student *t* distribution.

d mHealth: mobile health.

The results showed that none of the 5 covariates were identified as significant sources of heterogeneity in VO₂ peak. Each moderator was analyzed in a separate univariate random-effects meta-regression with the Hartung-Knapp-Sidik-Jonkman adjustment; for moderators with 3 categories (type of guidance and technology delivery platform), 2 pairwise contrasts were estimated relative to the reference category, and an Omnibus test was used for the joint effect of the moderator. None of the moderators reached statistical significance at the conventional 0.05 threshold: intervention duration (Omnibus *P*=.30), telemedicine mode (Omnibus *P*=.56), type of guidance (Omnibus *P*=.36), technology delivery platform (Omnibus *P*=.41), and intervention composition (Omnibus *P*=.44). The 95% CIs for all comparisons crossed 0, and the Omnibus tests showed no statistically significant moderator effect, suggesting that the moderating effect size of each variable cannot be determined based on the current evidence. Detailed coefficients for each comparison are shown in Table 2.

### Subgroup Analysis

Prespecified subgroup analyses were performed on the primary outcome VO₂ peak, and the results are summarized in Table 3. No statistically significant differences were found between groups in any of the subgroup analyses (all *P* values >.05), indicating that the existing evidence is insufficient to identify the aforementioned categorical variables as effect modifiers.

**Table 3.** Subgroup analysis of the effects of exercise-based telerehabilitation vs usual care on VO₂ peak (mL/kg/min) in patients with cardiovascular disease, based on 10 randomized controlled trials (n=749), stratified by 5 prespecified categorical moderators. Each subgroup was analyzed using a random-effects model with the HKSJ^a^ approach. No statistically significant between-group differences were detected for any moderator (all *P*>.05).

Moderator and group	Participants (I^b^/C^c^)	k^d^	Mean diff^e^	95% CI	*P* (overall)	*I*² (%)	*P* (group difference)
Duration of intervention							.30
≤12 weeks	190/181	6	3.33	1.85, 4.81	.002	37.13	
>12 weeks	197/181	4	1.92	−2.11, 5.96	.23	83.46	
Total	387/362	10	2.58	1.16, 4.00	.003	74.45	
Telemedicine mode							.56
Synchronous	170/167	5	2.24	−0.70, 5.19	.10	65.65	
Asynchronous	217/195	5	3.01	0.88, 5.14	.02	63.53	
Total	387/362	10	2.58	1.16, 4.00	.003	74.45	
Type of guidance							.36
Professional-led	162/154	5	3.02	1.14, 4.91	.01	31.85	
Patient self-managed	149/133	3	1.48	−5.87, 8.83	.48	81.90	
Technology-system-led	76/75	2	3.75	−2.73, 10.22	.09	9.18	
Total	387/362	10	2.58	1.16, 4.00	.003	74.45	
Technology platform							.41
Wearable sensor-based	102/81	3	2.26	−4.49, 9.01	.29	60.72	
Smartphone or mHealth^f^-based	191/183	5	3.51	2.21, 4.81	.002	31.41	
SMS-, web-, or telephone-based	94/98	2	1.75	−28.91, 32.40	.60	73.39	
Total	387/362	10	2.58	1.16, 4.00	.003	74.45	
Intervention composition							.44
Exercise-only	67/68	2	3.51	−4.55, 11.56	.11	6.16	
Multicomponent	320/294	8	2.34	0.59, 4.10	.02	78.08	
Total	387/362	10	2.58	1.16, 4.00	.003	74.45	

a HKSJ: Hartung-Knapp-Sidik-Jonkman.

b I: number of intervention group participants.

c C: number of control group participants.

d k: number of studies.

e Mean diff: pooled mean difference (mL/kg/min).

f mHealth: mobile health.

Regarding intervention duration, numerical differences were found between the effect estimates of the ≤12-week subgroup (n=371, MD 3.33, 95% CI 1.85‐4.81, *P*=.002, *I*²=37.13%) and the >12-week subgroup (n=378, MD 1.92, 95% CI −2.11 to 5.96, *P*=.23, *I*²=83.46%), with the latter’s CI crossing 0. The difference between groups was not statistically significant (*P*=.30). It is noteworthy that the >12-week subgroup exhibited extremely high heterogeneity (*I*²=83.46%). Given the limited number of studies within this subgroup and insufficient statistical power, this result should not be interpreted as a lack of effect with longer intervention durations.

Regarding telemedicine mode, the asynchronous mode subgroup (n=412, MD 3.01, 95% CI 0.88‐5.14, *P*=.02, *I*²=63.53%) showed a statistically significant effect, whereas the synchronous mode subgroup (n=337, MD 2.24, 95% CI −0.70 to 5.19, *P*=.10, *I*²=65.65%) did not reach statistical significance. Both subgroups exhibited moderate to high heterogeneity, and the effect estimates were of similar direction and magnitude. The between-subgroup difference was not statistically significant (*P*=.56), and the nonsignificant result in the synchronous subgroup should not be interpreted as evidence of absence of effect, given the relatively small number of contributing trials and the wide CI.

Regarding guidance type, the professional-led subgroup (n=316, MD 3.02, 95% CI 1.14‐4.91, *P*=.01, *I*²=31.85%) showed a statistically significant effect with low heterogeneity. The technology-system-led subgroup (n=151, MD 3.75, 95% CI −2.73 to 10.22, *P*=.09, *I*²=9.18%) yielded a numerically larger effect estimate but did not reach statistical significance, reflecting the very small number of contributing trials (*k*=2) and consequent imprecision rather than absence of effect. The patient self-managed subgroup (n=282, MD 1.48, 95% CI −5.87 to 8.83, *P*=.48) also showed a nonsignificant result with extremely wide CIs and very high within-subgroup heterogeneity (*I*²=81.90%), further limiting interpretability. The between-subgroup difference was not statistically significant (*P*=.36).

Regarding technology delivery platform, the smartphone or mHealth app subgroup (n=374, MD 3.51, 95% CI 2.21‐4.81, *P*=.002, *I*²=31.41%) showed accurate and statistically significant effect estimates with low heterogeneity within the subgroup. The CIs for the wearable sensor subgroup (n=183, MD 2.26, 95% CI −4.49 to 9.01, *P*=.29, *I*²=60.72%) and the SMS-, web-, or telephone-based subgroup (n=192, MD 1.75, 95% CI −28.91 to 32.40, *P*=.60, *I*²=73.39%) were extremely wide and crossed 0, reflecting high within-subgroup heterogeneity and insufficient statistical power due to the very small number of contributing trials (k=3 and k=2, respectively), rather than necessarily indicating ineffective intervention. The between-subgroup difference was not statistically significant (*P*=.41).

Regarding intervention composition, the multicomponent intervention subgroup (n=614, MD 2.34, 95% CI 0.59‐4.10, *P*=.02, *I*²=78.08%) showed a statistically significant effect, although with high heterogeneity, suggesting substantial variability in intervention effects within this subgroup. The exercise-only subgroup (n=135, MD 3.51, 95% CI −4.55 to 11.56, *P*=.11, *I*²=6.16%) yielded a numerically larger effect estimate but did not reach statistical significance, owing to the very small number of contributing trials (k=2) and the resulting wide CI, rather than to an absence of effect. The between-subgroup difference was not statistically significant (*P*=.44).

In summary, no statistically significant between-group effect modifications were detected in any subgroup analysis, and the existing evidence is insufficient to draw definitive conclusions regarding any single moderating variable.

### Publication Bias

**Figure 7.**
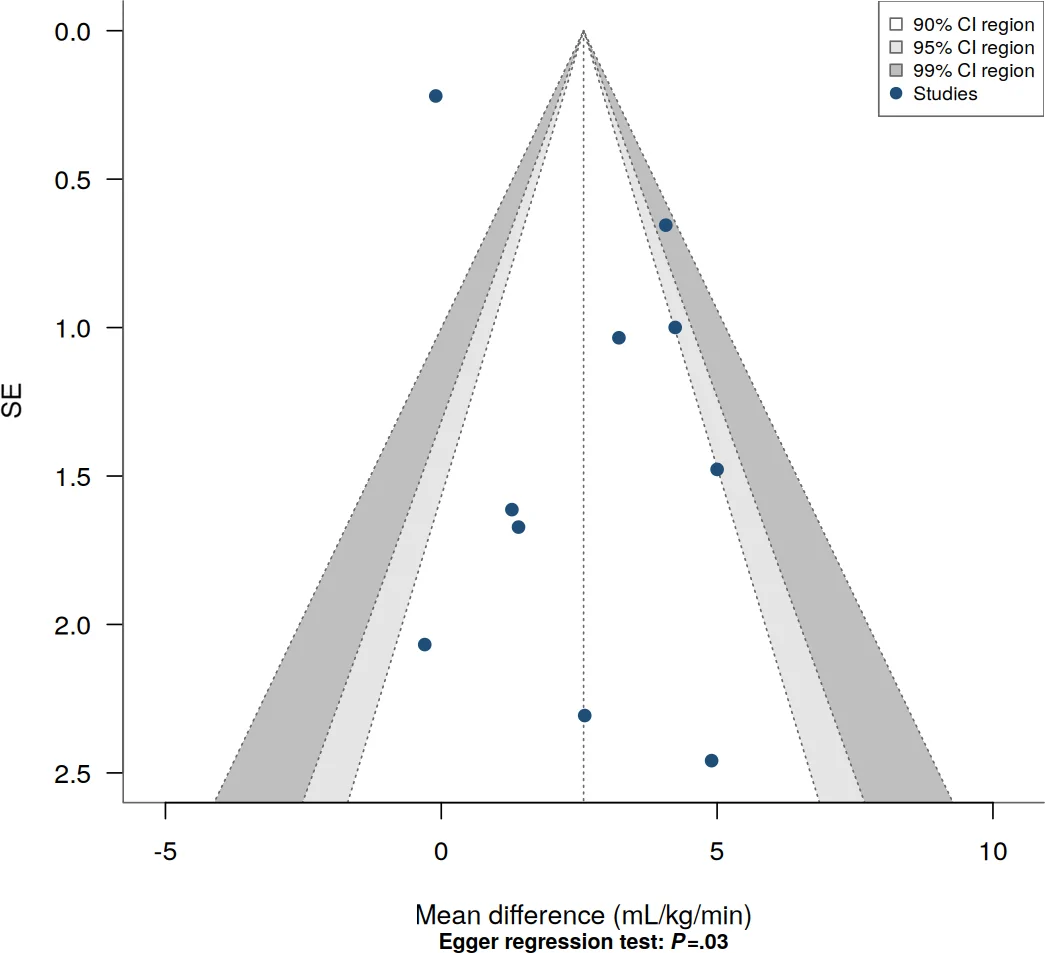
Funnel plot assessing publication bias for VO₂ peak outcomes from 10 randomized controlled trials of exercise-based telerehabilitation in patients with cardiovascular disease. Egger linear regression test indicated statistically significant funnel plot asymmetry (P=.03), suggesting a possible small sample effect that should be considered when interpreting the pooled results.

According to the Cochrane guidelines, the Egger linear regression test was used to assess small sample effects for outcome measures with ≥10 included studies. The Egger test result for the VO₂ peak was statistically significant (*P*=.03). As shown in Figure 7, the precision-standardized effect size scatter plot exhibited some asymmetry, and the lower bound of the 95% CI of the regression line intercept was close to but did not completely exclude 0, suggesting a possible small sample effect. The analyses of SBP and DBP each included 5 studies, which did not meet the minimum sample size requirement (≥10 studies) for publication bias testing. Therefore, no statistical tests were performed on these outcomes. However, the potential impact of publication bias should still be considered when the number of included studies is limited.

### Sensitivity Analysis

**Figure 8.**
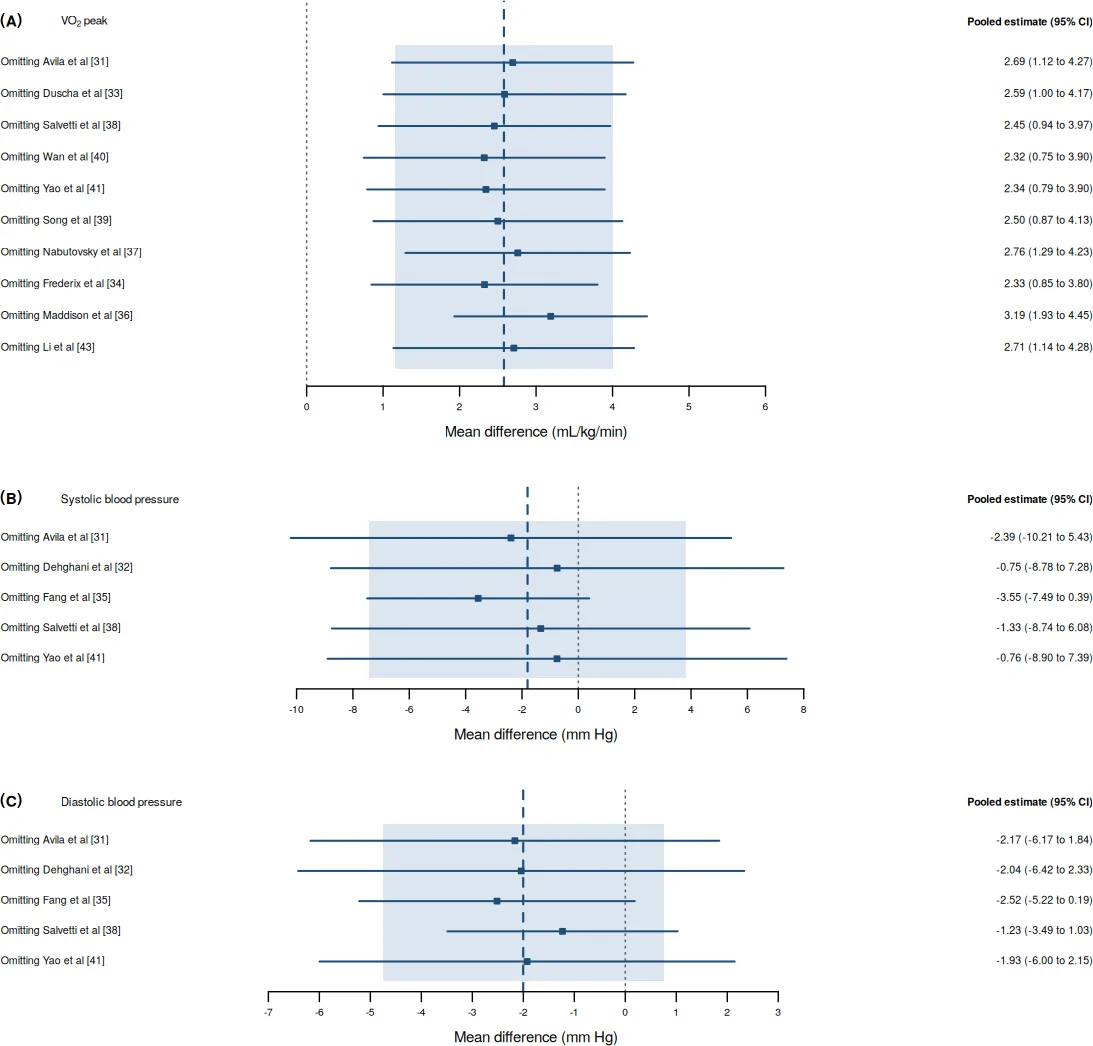
Leave-one-out sensitivity analysis for the 3 pooled outcomes. Each row shows the pooled mean difference and its 95% CI when the named trial is omitted. (A) VO₂ peak, 10 randomized controlled trials; (B) systolic blood pressure, 5 trials; (C) diastolic blood pressure, 5 trials. The dashed line marks the overall pooled estimate and the shaded band its 95% CI; the dotted line marks the line of no effect. All analyses use a random-effects model with the Sidik-Jonkman estimator and the Knapp-Hartung adjustment, as in Figures 4-6 [31-41,43].

Sensitivity analysis was performed on all outcome measures included in the meta-analysis, with the results shown in Figure 8 [31-41,43].

Regarding VO₂ peak (Figure 8A), after sequentially removing individual studies, the estimated pooled effect ranged from 2.32 to 3.19 mL/kg/min. The effect direction remained consistent across all removal scenarios, and the CIs did not cross 0, indicating that the pooled VO₂ peak results were not excessively influenced by any individual study and exhibited good robustness.

Regarding SBP (Figure 8B), after sequentially removing individual studies, the estimated pooled effect ranged from −3.55 to −0.75 mm Hg. The CIs crossed 0 across all removal scenarios, consistent with the overall pooled result, indicating that the pooled SBP results remained stable under sequential removal testing.

Regarding DBP (Figure 8C), after successively eliminating individual studies, the estimated pooled effect ranged from −2.52 to −1.23 mm Hg. The CIs for all elimination scenarios crossed 0, consistent with the overall pooled results, suggesting that the pooled DBP results are also relatively robust.

In summary, the sensitivity analyses of each of the 3 outcomes after elimination did not find any individual study that had a decisive impact on the pooled results, and the estimated pooled effect remained relatively consistent across all elimination scenarios.

### Certainty of Evidence

The GRADE framework was used to assess the certainty of evidence for the 3 outcomes (Table 4). For VO₂ peak, the evidence was rated as very low certainty due to serious concerns regarding inconsistency, indirectness, and imprecision. For SBP, the evidence was also rated as very low certainty owing to serious inconsistency, indirectness, and imprecision. For DBP, the evidence was rated as very low certainty, primarily driven by very serious indirectness and serious imprecision. Given the very low certainty of evidence across all 3 outcomes, the pooled effect estimates should be interpreted with caution. Detailed GRADE assessment procedures and justifications for each downgrade are provided in Section S4 in Multimedia Appendix 1.

**Table 4.** Overall certainty of evidence assessed using the GRADE^a^ approach for the effects of telemedicine-based exercise rehabilitation vs routine medical care on peak oxygen uptake, systolic blood pressure, and diastolic blood pressure in patients with cardiovascular disease.

	Certainty assessment							Patients, n		Effect		Certainty	Importance
	Studies, n	Study design	Risk of bias	Inconsistency	Indirectness	Imprecision	Other considerations	Telerehabilitation with exercise as the core component	Usual care or without structured exercise training	Relative (95% CI)	Absolute (95% CI)		
Peak oxygen uptake	10	Randomized trials	Not serious	Serious^b^	Serious^c^	Serious^d^	Publication bias strongly suspected	387	362	—^e^	MD^f^ 2.58 higher (1.16 higher to 4.00 higher)	⨁◯◯◯^g^ Very low	Critical
Systolic blood pressure	5	Randomized trials	Not serious	Serious^h^	Serious^i^	Serious^j^	Publication bias strongly suspected	148	147	—	MD 1.80 lower (7.42 lower to 3.81 higher)	⨁◯◯◯ Very low	Important
Diastolic blood pressure	5	Randomized trials	Not serious	Not serious	Very serious^k^	Serious^l^	Publication bias strongly suspected	148	147	—	MD 2.00 lower (4.76 lower to 0.75 higher)	⨁◯◯◯ Very low	Important

a GRADE: Grading of Recommendations, Assessment, Development, and Evaluation.

b Downgraded one level for inconsistency: substantial statistical heterogeneity (*I*²=74.45%, Q(9)=70.77, *P*<.001), with effect estimates in inconsistent directions across trials.

c Downgraded one level for indirectness: the included trials enrolled a heterogeneous mix of cardiovascular disease subtypes; exercise was embedded in multicomponent packages in 8 of the 10 trials contributing to this outcome; and VO₂ peak is a surrogate endpoint, with no trial reporting mortality, myocardial infarction, or hospitalization.

d Downgraded one level for imprecision: the 95% prediction interval (−1.28 to 6.44 mL/kg/min) crosses the null, so a null or slightly negative effect cannot be excluded in some clinical settings.

e Not available.

f MD: mean difference.

g Certainty of evidence (GRADE): the number of filled circles indicates the certainty rating: 4 filled circles, high; 3 filled and 1 open, moderate; 2 filled and 2 open, low; 1 filled and 3 open, very low.

h Downgraded one level for inconsistency: moderate heterogeneity (*I*²=58.24%, Q(4)=7.09, *P*=.13), with effect estimates in both directions.

i Downgraded one level for indirectness: only 1 of the 5 trials enrolled patients with hypertension and targeted blood pressure reduction; in the remaining trials, blood pressure was an incidentally measured secondary outcome, and delivery modes ranged from text messaging to AI-guided exercise prescription.

j Downgraded 1 level for imprecision: the 95% CI (−7.42 to 3.81 mm Hg) spans both clinically important benefit and harm, and is based on 295 participants.

k Downgraded 2 levels for indirectness: only 1 of the 5 trials enrolled patients with hypertension and targeted blood pressure reduction, and the interventions differed substantially in delivery mode, so the pooled estimate does not apply directly to any single target population.

l Downgraded 1 level for imprecision: the 95% CI (−4.76 to 0.75 mm Hg) crosses the null, and 295 participants are insufficient for a reliable estimate of this outcome.

## Discussion

### Principal Findings

This systematic review and meta-analysis of 13 RCTs examined the effects of exercise-based telerehabilitation on cardiometabolic outcomes in patients with CVD. Compared with usual care, exercise-based telerehabilitation produced a statistically significant improvement in VO₂ peak, but did not produce statistically significant changes in either SBP or DBP. Crucially, although the average effect on VO₂ peak was favorable, the 95% prediction interval crossed 0, indicating that in some future clinical settings the true effect could plausibly be null or even slightly negative. The pooled estimate should therefore be interpreted as the average effect across the included trial contexts rather than a universal effect that will be reproduced in every implementation [49,50]. Heterogeneity for VO₂ peak was substantial, and GRADE certainty was rated low to very low for VO₂ peak and very low for both blood pressure outcomes, reflecting concerns related to inconsistency, indirectness, imprecision, and possible publication bias [51]. Within this evidentiary frame, our findings support a probable, but not certain, beneficial effect of exercise-based telerehabilitation on cardiorespiratory fitness (CRF), and provide no convincing evidence of an antihypertensive effect at the pooled level.

To explore the substantial heterogeneity observed for VO₂ peak, we conducted both meta-regression and prespecified subgroup analyses across 5 candidate moderators (intervention duration, telemedicine mode, type of guidance, technology delivery platform, and intervention composition). None of these covariates explained a statistically significant proportion of the between-study variance, and no statistically significant between-group differences were detected in any subgroup contrast. The residual heterogeneity is therefore likely attributable to factors that we were unable to model directly, including intertrial variation in baseline CRF, exercise prescription parameters (intensity, weekly frequency, session duration, and progression), supervision intensity within nominally similar delivery modes, comorbidity burden, concurrent pharmacotherapy, and outcome assessment protocols (cardiopulmonary exercise testing vs estimated VO₂ peak from submaximal testing) [52,53]. Patient-level moderators such as age, sex, baseline functional capacity, and engagement with the digital platform are also plausible contributors [54]. We were unable to perform additional moderator analyses on these characteristics because individual participant data were not available, and aggregate-level reporting of exercise dose and adherence was inconsistent and frequently incomplete across the included studies. This limitation is acknowledged below and represents an important target for future trials.

### Comparison With Prior Work

VO₂ peak is the gold-standard index of CRF and a robust independent predictor of cardiovascular and all-cause mortality, with the American Heart Association recommending its use as a clinical vital sign [55,56]. A pooled improvement in the order of 2‐3 mL/kg/min, even at a population-average level, is therefore clinically meaningful, since each 1 metabolic equivalent (≈3.5 mL/kg/min) increment in CRF is associated with an approximately 10%-25% reduction in mortality risk in CVD populations [55]. Our pooled estimate is broadly consistent with that of Zhang and Lin [57], who reported a long-term VO₂ peak benefit of cardiac telerehabilitation vs center-based CR in patients with CAD, and aligns with the direction of effect reported in the network meta-analysis by Li et al [19] and the heart-failure-specific synthesis of Gao et al [21]. Mechanistically, exercise-induced gains in VO₂ peak are supported by well-established physiological adaptations including expanded blood volume, increased hemoglobin mass and capillary density, mitochondrial biogenesis, and improved cardiac output and peripheral oxygen extraction [58-60]. The convergence of our findings with these prior reviews suggests that a remotely delivered exercise stimulus, when adequately prescribed, can engage the same physiological pathways that underpin the benefits of center-based CR [61].

Our null findings for both SBP and DBP stand in apparent contrast to the well-documented antihypertensive effect of structured exercise training, where moderate-intensity aerobic, dynamic resistance, and isometric exercise have all been shown to lower resting blood pressure by 3‐7 mm Hg in hypertensive populations through improvements in endothelial function, reductions in sympathetic outflow, enhanced parasympathetic tone, and reduced peripheral vascular resistance [62,63]. However, our null pooled effect is in close agreement with previous telerehabilitation-focused syntheses: Cruz-Cobo et al [20] reported nonsignificant effects on both SBP (*P*=.99) and DBP (*P*=.36) after mHealth-delivered secondary prevention; Yu et al [27] reported a nonsignificant effect on SBP only; and Zhong et al [15] likewise reported no significant impact on cardiovascular risk factors including blood pressure. Several explanations are plausible. First, the included trials were not designed primarily to lower blood pressure: most enrolled normotensive or pharmacologically controlled participants in whom further reductions are mechanistically constrained [64,65], and only 1 trial explicitly enrolled patients with essential hypertension [41]. Second, the exercise dose actually delivered through remote channels is often below the prescribed dose, and adherence in unsupervised settings tends to decline over time, weakening the hemodynamic stimulus [66,67]. Third, the small number of contributing studies (n=5 for each blood pressure outcome) and a pooled sample of 295 participants leave the analyses underpowered to detect modest effects. The nurse-led telerehabilitation meta-analysis by Lee et al [68] demonstrated that statistically significant SBP reductions are achievable in disease-targeted populations (MD 10.48 mm Hg in hypertension and diabetes subgroups), suggesting that null pooled effects in mixed CVD populations are likely population- and dose-specific rather than evidence of biological inefficacy. The current evidence therefore neither establishes nor refutes an antihypertensive effect of exercise-based telerehabilitation, and dedicated trials in hypertensive cohorts are needed.

### Limitations

#### Conceptual Heterogeneity of the Included Interventions

Although our review prespecified “exercise-based telerehabilitation” as the intervention of interest, 8 of the 13 included trials embedded the exercise prescription within a broader package that also included structured health education, dietary counseling, behavior-change techniques, or psychological support, while only 2 trials delivered exercise as a sole intervention. This is a recurring problem across the wider telerehabilitation literature: prior reviews by Yang et al [26], Yu et al [27], Ramachandran et al [23], and Cruz-Cobo et al [20] similarly bundled exercise with multidisciplinary lifestyle components. As a consequence, the pooled effects reported here, particularly for VO₂ peak, cannot be unambiguously attributed to the exercise stimulus alone, and could partly reflect concurrent improvements in self-management, dietary intake, medication adherence, or motivational support delivered through the same digital platform [69,70]. Reassuringly, our prespecified subgroup analysis showed that the exercise-only subgroup produced a numerically larger and more homogeneous estimate than the multicomponent subgroup, but the limited number of exercise-only trials prevents firm causal inference. We have therefore framed the conclusions as effects of exercise-based telerehabilitation programs as currently delivered, rather than as effects of exercise per se, and we identify component-isolated trials as a priority for future work.

#### Digital Health–Specific Considerations

As telerehabilitation is fundamentally a digitally mediated intervention, its effectiveness is shaped not only by the prescribed exercise dose but also by adherence, engagement, usability, and the specific characteristics of the delivery technology—domains that conventional center-based CR research does not need to consider in the same way [71,72]. The included trials reported these dimensions inconsistently: only a minority quantified session-level adherence, fewer reported continuous engagement metrics (eg, app-opens and sensor wear-time), and very few reported usability scores or drop-off curves. This omission matters substantially. Olivier et al [73] documented how a virtual CR trial was prematurely terminated because of poor app product quality, low smartphone compatibility, and inadequate technology support, despite a sound clinical concept. The same review identified inexperienced technology partners and poor product design as systemic risks for digital health trials. Our subgroup findings, in which smartphone or mHealth platforms produced statistically significant and highly homogeneous improvements in VO₂ peak while wearable-sensor and SMS-, web-, or telephone-based platforms produced wider, nonsignificant pooled estimates with greater heterogeneity, are best interpreted as preliminary signals that platform characteristics may modify intervention effectiveness, but the analyses are underpowered and cannot identify which specific platform attributes (interactive feedback frequency, push-notification design, data visualization, or ease of pairing with sensors) drive the differences [74,75]. Likewise, the larger and more homogeneous effect observed in the professional-led and technology-system-led guidance subgroups, contrasted with the wide and nonsignificant interval in the patient-self-managed subgroup, is consistent with broader evidence that human or intelligent real-time support enhances self-efficacy, accountability, and exercise adherence in remote settings. Likewise, the larger pooled effect estimates observed in both the professional-led and technology-system-led guidance subgroups, contrasted with the patient-self-managed subgroup, are consistent with broader evidence that human or intelligent real-time support enhances self-efficacy, accountability, and exercise adherence in remote settings [57]. We note, however, that the technology-system-led subgroup contained only 2 trials and yielded a wide CI that did not exclude the null; this pattern should be regarded as hypothesis-generating rather than confirmatory, and adequately powered head-to-head trials are needed to establish whether intelligent system-led delivery confers an effect comparable to that of professional-led delivery. Future telerehabilitation trials should therefore report adherence and engagement using standardized digital-health metrics, examine usability and acceptability with validated instruments such as the System Usability Scale, mHealth App Usability Questionnaire [76], and stratify reporting by user demographics to ensure findings translate to digitally less-engaged subpopulations [77].

#### Cautious Interpretation of Subgroup Findings

Several subgroup contrasts produced nonsignificant pooled estimates with CIs that crossed 0, including the >12-week duration subgroup, the synchronous-mode subgroup, the patient self-managed and technology-system-led guidance subgroups, the wearable-sensor and SMS-, web-, or telephone-based platform subgroups, and the exercise-only composition subgroup. These results should not be interpreted as evidence that longer-duration, self-managed, or nonapp-based telerehabilitation is ineffective. Each of these subgroups contained a small number of studies, exhibited high within-subgroup heterogeneity, and was therefore underpowered to detect plausible effect sizes [78]. None of the between-group difference tests reached statistical significance (all *P*>.05), meaning that the apparent contrast between subgroups is itself uncertain. The conventional caution that absence of evidence is not evidence of absence applies particularly strongly to underpowered subgroup contrasts in meta-analyses with limited primary studies [79]. Accordingly, the pattern observed should be regarded as hypothesis-generating, and adequately powered head-to-head trials are required before any subgroup of telerehabilitation can be deprioritized on efficacy grounds.

The certainty of the evidence underpinning our findings is limited in several ways. First, with respect to risk of bias, 53.8% (7/13) of the included trials were judged to be at high overall risk of bias, and a further 30.8% (4/13) raised some concerns, driven primarily by inadequate description of randomization procedures, lack of allocation concealment, and absence of preregistration of primary outcomes [80]. Blinding of participants and providers was not feasible given the nature of the intervention, but the resulting performance bias may have inflated effect estimates, particularly for outcomes with a perceived behavioral component. Second, with respect to inconsistency, between-study heterogeneity was substantial for VO₂ peak and moderate for SBP, and was not explained by any of the 5 prespecified moderators. The wide 95% prediction interval for VO₂ peak underscores that the average effect should not be assumed to apply to every clinical setting [49,50]. Third, with respect to indirectness, the included populations spanned a broad CVD spectrum (CAD, post–percutaneous coronary intervention, ischemic heart disease, heart failure, hypertension, low-risk coronary patients, and a pediatric Congenital Heart Disease cohort), and the interventions varied in exercise mode, dose, duration, supervision, and digital platform, limiting the directness of the evidence to any specific patient or program configuration. Fourth, with respect to imprecision, the blood pressure analyses included only 5 trials each and 295 participants in total, and the CIs were wide enough to encompass clinically meaningful effects in either direction. Fifth, the Egger regression test indicated possible small-study effects for VO₂ peak, and the small number of trials precluded formal assessment for the blood pressure outcomes. Sixth, the included studies provided limited or inconsistent data on several clinically important implementation dimensions. Adherence to the prescribed intervention was reported in only 10 of 13 trials, and even among those, the metric used varied substantially—ranging from exercise sessions per week and daily step counts to platform login frequency, message engagement rates, and percentage of prescribed sessions completed—precluding any pooled estimate of adherence or its use as a quantitative moderator of efficacy [81]. Adverse event surveillance was similarly inconsistent: although 10 of 13 trials included an explicit safety statement, 3 trials [33-35] provided no dedicated adverse event reporting whatsoever, and the 9 trials that declared no serious exercise-related adverse events did so without describing a prespecified surveillance protocol, making it difficult to distinguish genuine safety from inadequate monitoring [82]. No trial reported data on cost-effectiveness, and patient experience or usability was addressed in only a small minority of studies [83]. Long-term follow-up beyond the active intervention period was absent in all included trials, leaving the durability of the observed CRF gains unknown [84]. Collectively, these gaps restrict any comprehensive judgment of the real-world value and implementation readiness of exercise-based telerehabilitation, and they underscore the need for future trials to adopt standardized reporting of adherence, engagement, safety surveillance, and health-economic outcomes.

For clinical practice, the present evidence supports the cautious adoption of exercise-based telerehabilitation as an option for improving CRF in patients with CVD, particularly when integrated with professional or intelligent-system support and delivered via well-designed mobile platforms [85]. However, given the low to very low GRADE certainty and the wide prediction interval for VO₂ peak, telerehabilitation should be presented as an alternative pathway for patients who cannot access center-based CR, rather than as a uniformly equivalent substitute. For health policy, investment in telerehabilitation should be accompanied by quality-assurance frameworks specifying minimum reporting standards for exercise dose, adherence, engagement, and adverse events, and should incorporate equity considerations to ensure that digitally less-engaged populations are not systematically excluded [86,87]. For future research, 3 priorities emerge. First, component-isolated trials are needed that compare exercise-only telerehabilitation against multicomponent telerehabilitation to disentangle the contribution of the exercise stimulus from concurrent behavioral and educational elements [88]. Second, dedicated trials in hypertensive cohorts are needed to clarify whether telerehabilitation can produce clinically meaningful blood-pressure reductions when adequately powered and dose-targeted. Third, telerehabilitation trials should adopt standardized digital health reporting, capturing engagement and usability with validated instruments, reporting platform-level retention curves, and conducting embedded health-economic and patient-experience evaluations to inform real-world implementation [89,90].

### Conclusions

This systematic review and meta-analysis demonstrates that exercise-based telerehabilitation, compared with usual care, produces a probable improvement in CRF in adults with CVD, while providing no convincing evidence of an antihypertensive effect at the pooled level. The strength of these conclusions is tempered by substantial between-study heterogeneity, low to very low GRADE certainty, and a wide prediction interval for VO₂ peak that admits the possibility of null or slightly negative effects in some implementation contexts. None of the 5 prespecified digital health–specific moderators—intervention duration, telemedicine mode, type of guidance, technology delivery platform, or intervention composition—statistically explained the observed heterogeneity, although exploratory subgroup patterns suggest that smartphone- or mHealth-app-based delivery and professional- or technology-system-led guidance may be associated with more favorable and more consistent gains in CRF; these patterns should be regarded as hypothesis-generating rather than definitive.

Taken together, our findings position exercise-based telerehabilitation as a clinically promising but evidence immaturity–related alternative or adjunct to center-based CR. The most consequential gaps in the existing literature are the bundling of exercise with multiple nonexercise components, the underreporting of digital health specific implementation metrics (adherence, engagement, usability, or retention curves), the absence of long-term follow-up, and the lack of dedicated trials in hypertensive cohorts. Resolving these gaps will require component-isolated trials, standardized digital-health reporting, embedded health-economic and patient-experience evaluations, and equity-conscious implementation strategies. Until such evidence is available, exercise-based telerehabilitation should be offered as a patient-centered alternative pathway for individuals who cannot access center-based CR, rather than as a uniformly equivalent substitute, and clinicians, program designers, and policymakers should treat the cardiometabolic effects reported here as an average signal across heterogeneous implementations rather than as a guaranteed effect for every patient and every platform.

## Supplementary material

10.2196/95923 Multimedia Appendix 1 Database-specific search strategies, citation overlap matrix used to calculate the corrected covered area, per-study adverse event and adherence reporting, and detailed GRADE (Grading of Recommendations, Assessment, Development, and Evaluation) assessment procedures and justifications.

10.2196/95923 Checklist 1 PRISMA 2020 checklist.

## Abbreviations

CAD: coronary artery disease
CCA: corrected covered area
CR: cardiac rehabilitation
CRF: cardiorespiratory fitness
CVD: cardiovascular disease
DBP: diastolic blood pressure
GRADE: Grading of Recommendations, Assessment, Development, and Evaluation
MD: mean difference
PRISMA: Preferred Reporting Items for Systematic Reviews and Meta-Analyses
PRISMA-S: Preferred Reporting Items for Systematic Reviews and Meta-Analyses literature search extension
PROSPERO: International Prospective Register of Systematic Reviews
RCT: randomized controlled trial
RoB 2: Cochrane Risk of Bias Tool version 2
SBP: systolic blood pressure
VO₂ peak: peak oxygen uptake
WHO: World Health Organization
