# Supplementary material for: Effects of Telerehabilitation With Exercise as the Core Component on Peak Oxygen Uptake and Blood Pressure in Patients With Cardiovascular Disease: Systematic Review and Meta-Analysis
Source: J Med Internet Res. 2026 Sep 21;28:e95923. doi: 10.2196/95923 (PMC13592150; doi:10.2196/95923)
Supplement: Supplementary file 1 [file jmir-v28-e95923-s001.docx]

# Section S1 — Search Strategy

Table S1.1 Pubmed 220

| Search Number | Query | Results |
| --- | --- | --- |
| #1 | "Myocardial Ischemia"[MeSH Terms] | 498,191 |
| #2 | "heart disease ischemic"[Title/Abstract] OR "disease ischemic heart"[Title/Abstract] OR "diseases ischemic heart"[Title/Abstract] OR "heart diseases ischemic"[Title/Abstract] OR "ischemic heart diseases"[Title/Abstract] OR "ischemia myocardial"[Title/Abstract] OR "ischemias myocardial"[Title/Abstract] OR "myocardial ischemias"[Title/Abstract] OR "ischemic heart disease"[Title/Abstract] | 35,190 |
| #3 | "Coronary Artery Disease"[MeSH Terms] | 85049 |
| #4 | "artery disease coronary"[Title/Abstract] OR "artery diseases coronary"[Title/Abstract] OR "coronary artery diseases"[Title/Abstract] OR "coronary arteriosclerosis"[Title/Abstract] OR (("Arteriosclerosis"[MeSH Terms] OR "Arteriosclerosis"[All Fields] OR "Arterioscleroses"[All Fields]) AND "Coronary"[Title/Abstract]) OR (("coronaries"[All Fields] OR "heart"[MeSH Terms] OR "heart"[All Fields] OR "Coronary"[All Fields]) AND "Arterioscleroses"[Title/Abstract]) OR "arteriosclerosis coronary"[Title/Abstract] OR "atherosclerosis coronary"[Title/Abstract] OR (("Atherosclerosis"[MeSH Terms] OR "Atherosclerosis"[All Fields] OR "Atheroscleroses"[All Fields]) AND "Coronary"[Title/Abstract]) OR (("coronaries"[All Fields] OR "heart"[MeSH Terms] OR "heart"[All Fields] OR "Coronary"[All Fields]) AND "Atheroscleroses"[Title/Abstract]) OR "coronary atherosclerosis"[Title/Abstract] OR "left main coronary artery disease"[Title/Abstract] OR "left main coronary disease"[Title/Abstract] OR "left main disease"[Title/Abstract] OR "left main diseases"[Title/Abstract] | 111412 |
| #5 | "Acute Coronary Syndrome"[MeSH Terms] | 22658 |
| #6 | "acute coronary syndromes"[Title/Abstract] OR "coronary syndrome acute"[Title/Abstract] OR "coronary syndromes acute"[Title/Abstract] OR "syndrome acute coronary"[Title/Abstract] OR "syndromes acute coronary"[Title/Abstract] | 15546 |
| #7 | "Myocardial Infarction"[MeSH Terms] | 205159 |
| #8 | "infarction myocardial"[Title/Abstract] OR "infarctions myocardial"[Title/Abstract] OR "myocardial infarctions"[Title/Abstract] OR "heart attack"[Title/Abstract] OR "heart attacks"[Title/Abstract] OR "myocardial infarct"[Title/Abstract] OR "infarct myocardial"[Title/Abstract] OR "infarcts myocardial"[Title/Abstract] OR "myocardial infarcts"[Title/Abstract] OR "cardiovascular stroke"[Title/Abstract] OR "cardiovascular strokes"[Title/Abstract] OR "stroke cardiovascular"[Title/Abstract] OR "strokes cardiovascular"[Title/Abstract] | 36432 |
| #9 | "Angina Pectoris"[MeSH Terms] | 45434 |
| #10 | "angor pectoris"[Title/Abstract] OR "Stenocardia"[Title/Abstract] OR "Stenocardias"[Title/Abstract] | 960 |
| #11 | "Percutaneous Coronary Intervention"[MeSH Terms] | 71202 |
| #12 | "coronary intervention percutaneous"[Title/Abstract] OR "coronary interventions percutaneous"[Title/Abstract] OR "intervention percutaneous coronary"[Title/Abstract] OR "interventions percutaneous coronary"[Title/Abstract] OR "percutaneous coronary interventions"[Title/Abstract] OR "percutaneous coronary revascularization"[Title/Abstract] OR "coronary revascularization percutaneous"[Title/Abstract] OR "coronary revascularizations percutaneous"[Title/Abstract] OR "percutaneous coronary revascularizations"[Title/Abstract] OR "revascularization percutaneous coronary"[Title/Abstract] OR "revascularizations percutaneous coronary"[Title/Abstract] | 6034 |
| #13 | "Coronary Artery Bypass"[MeSH Terms] | 58935 |
| #14 | "artery bypass coronary"[Title/Abstract] OR ((("arterialization"[All Fields] OR "arterializations"[All Fields] OR "arterialize"[All Fields] OR "arterialized"[All Fields] OR "arterializing"[All Fields] OR "arterially"[All Fields] OR "arterials"[All Fields] OR "arterie"[All Fields] OR "arteries"[MeSH Terms] OR "arteries"[All Fields] OR "arterial"[All Fields] OR "arteris"[All Fields] OR "Artery"[All Fields] OR "arterious"[All Fields] OR "artery s"[All Fields] OR "arterys"[All Fields]) AND ("Bypass"[All Fields] OR "bypassed"[All Fields] OR "Bypasses"[All Fields] OR "bypassing"[All Fields])) AND "Coronary"[Title/Abstract]) OR (("Bypass"[All Fields] OR "bypassed"[All Fields] OR "Bypasses"[All Fields] OR "bypassing"[All Fields]) AND "coronary artery"[Title/Abstract]) OR "coronary artery bypasses"[Title/Abstract] OR "coronary artery bypass grafting"[Title/Abstract] OR "coronary artery bypass surgery"[Title/Abstract] OR "aortocoronary bypass"[Title/Abstract] OR "aortocoronary bypasses"[Title/Abstract] OR "bypass aortocoronary"[Title/Abstract] OR (("Bypass"[All Fields] OR "bypassed"[All Fields] OR "Bypasses"[All Fields] OR "bypassing"[All Fields]) AND "Aortocoronary"[Title/Abstract]) OR "bypass surgery coronary artery"[Title/Abstract] OR "bypass coronary artery"[Title/Abstract] | 73781 |
| #15 | #1 OR #2 OR #3 OR #4 OR #5 OR #6 OR #7 OR #8 OR #9 OR #10 OR #11 OR #12 OR #13 OR #14 | 602226 |
| #16 | "Telemedicine"[MeSH Terms] | 57233 |
| #17 | "virtual medicine"[Title/Abstract] OR "medicine virtual"[Title/Abstract] OR "Tele-Referral"[Title/Abstract] OR "Tele-Referral"[Title/Abstract] OR "Tele-Referrals"[Title/Abstract] OR "mobile health"[Title/Abstract] OR "health mobile"[Title/Abstract] OR "mHealth"[Title/Abstract] OR "Telehealth"[Title/Abstract] OR "eHealth"[Title/Abstract] OR "tele intensive care"[Title/Abstract] OR "tele intensive care"[Title/Abstract] OR "Tele-ICU"[Title/Abstract] OR "Tele-ICU"[Title/Abstract] OR "Telecare"[Title/Abstract] OR "Tele-Care"[Title/Abstract] OR "Tele-Care"[Title/Abstract] | 52593 |
| #18 | "Randomized Controlled Trials as Topic"[MeSH Terms] OR "Randomized Controlled Trial"[Publication Type] OR "Controlled Clinical Trials as Topic"[MeSH Terms] | 857486 |
| #19 | "clinical trials randomized"[Title/Abstract] OR "trials randomized clinical"[Title/Abstract] OR "controlled clinical trials randomized"[Title/Abstract] | 258 |
| #20 | #16 OR #17 | 87193 |
| #21 | #18 OR #19 | 857669 |
| #22 | #20 AND #21 AND #15 | 210 |

Table S1.2 Embase 510

| **Search Number** | **Query** | **Results** |
| --- | --- | --- |
| **#1** | 'heart muscle ischemia'/exp | 111,970 |
| **#2** | 'acute heart muscle ischaemia':ab,ti OR 'acute heart muscle ischemia':ab,ti OR 'cardiac ischaemia':ab,ti OR 'cardiac ischemia':ab,ti OR 'cardiac muscle ischaemia':ab,ti OR 'cardiac muscle ischemia':ab,ti OR 'coronary artery ischaemia':ab,ti OR 'coronary artery ischemia':ab,ti OR 'coronary ischaemia':ab,ti OR 'coronary ischemia':ab,ti OR 'coronary syndrome':ab,ti OR 'heart anoxia':ab,ti OR 'heart hypoxia':ab,ti OR 'heart ischaemia':ab,ti OR 'heart ischaemic arrest':ab,ti OR 'heart ischaemic attack':ab,ti OR 'heart ischaemic time':ab,ti OR 'heart ischemia':ab,ti OR 'heart ischemic arrest':ab,ti OR 'heart ischemic attack':ab,ti OR 'heart ischemic time':ab,ti OR 'heart muscle hypoxia':ab,ti OR 'heart muscle ischaemia':ab,ti OR 'heart muscle ischaemia, subepicardial':ab,ti OR 'heart muscle ischemia, subepicardial':ab,ti OR 'heart transient ischaemic attack':ab,ti OR 'heart transient ischemic attack':ab,ti OR 'hypoxia, heart':ab,ti OR 'hypoxic heart':ab,ti OR 'ischaemic heart':ab,ti OR 'ischaemic heart arrest':ab,ti OR 'ischaemic myocardium':ab,ti OR 'ischemic heart':ab,ti OR 'ischemic heart arrest':ab,ti OR 'ischemic myocardium':ab,ti OR 'myocardial anoxia':ab,ti OR 'myocardial hypoxia':ab,ti OR 'myocardial ischaemia':ab,ti OR 'myocardial ischemia':ab,ti OR 'myocardium hypoxia':ab,ti OR 'myocardium ischaemia':ab,ti OR 'myocardium ischemia':ab,ti OR 'subendocardial ischaemia':ab,ti OR 'subendocardial ischemia':ab,ti OR 'transient ischaemic attack, heart':ab,ti OR 'transient ischemic attack, heart':ab,ti OR 'heart muscle ischemia':ab,ti | 198,512 |
| **#3** | 'coronary artery disease'/exp | 492,038 |
| **#4** | 'coronary disease':ab,ti OR 'multivessel coronary artery disease':ab,ti OR 'coronary artery disease':ab,ti | 205,068 |
| **#5** | 'acute coronary syndrome'/exp | 92,706 |
| **#6** | 'acute coronary syndromes':ab,ti OR 'acute coronary syndrome':ab,ti | 81,554 |
| **#7** | 'heart infarction'/exp | 540,484 |
| **#8** | 'cardiac infarct':ab,ti OR 'cardiac infarction':ab,ti OR 'cardial infarct':ab,ti OR 'heart attack':ab,ti OR 'heart infarct':ab,ti OR 'heart micro infarction':ab,ti OR 'heart muscle infarction':ab,ti OR 'infarction, heart':ab,ti OR 'myocardial infarct':ab,ti OR 'myocardial infarction':ab,ti OR 'myocardium infarct':ab,ti OR 'myocardium infarction':ab,ti OR 'premonitory infarction sign':ab,ti OR 'second heart attack':ab,ti OR 'subendocardial infarction':ab,ti OR 'transmural cardiac infarction':ab,ti OR 'transmural heart infarction':ab,ti OR 'transmural infarction, heart':ab,ti OR 'heart infarction':ab,ti | 387,206 |
| **#9** | 'angina pectoris'/exp | 129,262 |
| **#10** | 'angina':ab,ti OR 'anginal attack':ab,ti OR 'stenocardia':ab,ti OR 'angina pectoris':ab,ti | 93,419 |
| **#11** | 'percutaneous coronary intervention'/exp OR 'percutaneous coronary intervention' | 161,940 |
| **#12** | 'percutaneous coronary intervention':ab,ti | 82,562 |
| **#13** | 'coronary artery bypass graft'/exp OR 'coronary artery bypass graft' | 110,450 |
| **#14** | 'aorta coronary artery bypass':ab,ti OR 'aorta coronary bypass':ab,ti OR 'aorta coronary bypass graft':ab,ti OR 'aorta coronary vein bypass':ab,ti OR 'aorta coronary vein bypass graft':ab,ti OR 'aorta coronary vein shunt':ab,ti OR 'aortic coronary artery bypass':ab,ti OR 'aortic coronary bypass':ab,ti OR 'aorticocoronary anastomosis':ab,ti OR 'aorto coronary artery bypass':ab,ti OR 'aorto coronary bypass graft':ab,ti OR 'aorto coronary vein bypass':ab,ti OR 'aortocoronary anastomosis':ab,ti OR 'aortocoronary artery bypass':ab,ti OR 'aortocoronary artery bypass graft':ab,ti OR 'aortocoronary bypass':ab,ti OR 'aortocoronary bypass graft':ab,ti OR 'aortocoronary shunt':ab,ti OR 'aortocoronary vein bypass':ab,ti OR 'aortocoronary vein bypass graft':ab,ti OR 'aortocoronary venous bypass':ab,ti OR 'aortocoronary venous bypass graft':ab,ti OR 'coronary artery bypass':ab,ti OR 'coronary artery bypass grafting':ab,ti OR 'coronary artery graft':ab,ti OR 'coronary bypass':ab,ti OR 'coronary bypass graft':ab,ti OR 'coronary bypass grafting':ab,ti OR 'coronary vein bypass graft':ab,ti OR 'coronary venous bypass graft':ab,ti OR 'coronary artery bypass graft':ab,ti | 84,780 |
| **#15** | #1 OR #2 OR #3 OR #4 OR #5 OR #6 OR #7 OR #8 OR #9 OR #10 OR #11 OR #12 OR #13 OR #14 | 1,197,168 |
| **#16** | 'telemedicine'/exp OR 'telemedicine' | 109,988 |
| **#17** | 'tele medicine':ab,ti OR 'virtual medicine':ab,ti OR 'telemedicine':ab,ti | 35,098 |
| **#18** | #16 OR #17 | 110,015 |
| **#19** | 'randomized controlled trial'/exp OR 'randomized controlled trial' | 1,483,540 |
| **#20** | 'controlled trial, randomized':ab,ti OR 'randomised controlled study':ab,ti OR 'randomised controlled trial':ab,ti OR 'randomized controlled study':ab,ti OR 'trial, randomized controlled':ab,ti OR 'randomized controlled trial':ab,ti | 298,950 |
| **#21** | #19 OR #20 | 1,494,389 |
| **#22** | #15 AND #18 AND #21 | 510 |

Table S1.3 Web of Science 177

| **Search Number** | **Query** | **Results** |
| --- | --- | --- |
| **#1** | ((((((TS=(Myocardial Ischemia)) OR TS=(Coronary Artery Disease)) OR TS=(Acute Coronary Syndrome)) OR TS=(Myocardial Infarction)) OR TS=(Angina Pectoris)) OR TS=(Percutaneous Coronary Intervention)) OR TS=(Coronary Artery Bypass) | 528927 |
| **#2** | (TI=(“Disease*, Ischemic Heart” OR “Heart Diseases, Ischemic” OR “Ischemic Heart Diseases” OR “Ischemia*, Myocardial” OR “Myocardial Ischemias” OR “Ischemic Heart Disease” OR “Artery Disease*, Coronary” OR “Coronary Artery Diseases” OR “Coronary Arteriosclerosis” OR “Arterioscleroses, Coronary” OR “Coronary Arterioscleroses” OR “Arteriosclerosis*, Coronary” OR “Coronary Atheroscleros*” OR “Left Main Coronary Artery Disease” OR “Left Main Coronary Disease” OR “Left Main Disease*” OR “Acute Coronary Syndromes” OR “Coronary Syndrome*, Acute” OR “Syndrome*, Acute Coronary” OR “Infarction*, Myocardial” OR “Myocardial Infarctions” OR “Heart Attack*” OR “Myocardial Infarct” OR “Infarct*, Myocardial” OR “Myocardial Infarcts” OR “Cardiovascular Stroke*” OR “Stroke*, Cardiovascular” OR “Angor Pectoris” OR “Stenocardia*” OR “Coronary Intervention*, Percutaneous” OR “Intervention*, Percutaneous Coronary” OR “Percutaneous Coronary Interventions” OR “Percutaneous Coronary Revascularization” OR “Coronary Revascularization*, Percutaneous” OR “Percutaneous Coronary Revascularizations” OR “Revascularization*, Percutaneous Coronary” OR “Artery Bypass*, Coronary” OR “Bypasses, Coronary Artery” OR “Coronary Artery Bypasses” OR “Coronary Artery Bypass Grafting” OR “Coronary Artery Bypass Surgery” OR “Aortocoronary Bypass*” OR “Bypass*, Aortocoronary” OR “Bypass Surgery, Coronary Artery” OR “Bypass, Coronary Artery” OR “Heart Disease, Ischemic”)) OR AB=(“Disease*, Ischemic Heart” OR “Heart Diseases, Ischemic” OR “Ischemic Heart Diseases” OR “Ischemia*, Myocardial” OR “Myocardial Ischemias” OR “Ischemic Heart Disease” OR “Artery Disease*, Coronary” OR “Coronary Artery Diseases” OR “Coronary Arteriosclerosis” OR “Arterioscleroses, Coronary” OR “Coronary Arterioscleroses” OR “Arteriosclerosis*, Coronary” OR “Coronary Atheroscleros*” OR “Left Main Coronary Artery Disease” OR “Left Main Coronary Disease” OR “Left Main Disease*” OR “Acute Coronary Syndromes” OR “Coronary Syndrome*, Acute” OR “Syndrome*, Acute Coronary” OR “Infarction*, Myocardial” OR “Myocardial Infarctions” OR “Heart Attack*” OR “Myocardial Infarct” OR “Infarct*, Myocardial” OR “Myocardial Infarcts” OR “Cardiovascular Stroke*” OR “Stroke*, Cardiovascular” OR “Angor Pectoris” OR “Stenocardia*” OR “Coronary Intervention*, Percutaneous” OR “Intervention*, Percutaneous Coronary” OR “Percutaneous Coronary Interventions” OR “Percutaneous Coronary Revascularization” OR “Coronary Revascularization*, Percutaneous” OR “Percutaneous Coronary Revascularizations” OR “Revascularization*, Percutaneous Coronary” OR “Artery Bypass*, Coronary” OR “Bypasses, Coronary Artery” OR “Coronary Artery Bypasses” OR “Coronary Artery Bypass Grafting” OR “Coronary Artery Bypass Surgery” OR “Aortocoronary Bypass*” OR “Bypass*, Aortocoronary” OR “Bypass Surgery, Coronary Artery” OR “Bypass, Coronary Artery” OR “Heart Disease, Ischemic”) | 91457 |
| **#3** | #1 OR #2 | 548585 |
| **#4** | TS=(Telemedicine) | 44015 |
| **#5** | (TI=(“Virtual Medicine” OR “Medicine, Virtual” OR “Tele-Referral” OR “Tele Referral” OR “Tele-Referrals” OR “Mobile Health” OR “Health, Mobile” OR “mHealth” OR “Telehealth” OR “eHealth” OR “Tele-Intensive Care” OR “Tele Intensive Care” OR “Tele-ICU” OR “Tele ICU” OR “Telecare” OR “Tele-Care” OR “Tele Care”)) OR AB=(“Virtual Medicine” OR “Medicine, Virtual” OR “Tele-Referral” OR “Tele Referral” OR “Tele-Referrals” OR “Mobile Health” OR “Health, Mobile” OR “mHealth” OR “Telehealth” OR “eHealth” OR “Tele-Intensive Care” OR “Tele Intensive Care” OR “Tele-ICU” OR “Tele ICU” OR “Telecare” OR “Tele-Care” OR “Tele Care”) | 42159 |
| **#6** | #4 OR #5 | 78424 |
| **#7** | TS=(randomized controlled trial) | 609074 |
| **#8** | (TI=(“Clinical Trials, Randomized” OR “Trials, Randomized Clinical” OR “Controlled Clinical Trials, Randomized”)) OR AB=(“Clinical Trials, Randomized” OR “Trials, Randomized Clinical” OR “Controlled Clinical Trials, Randomized”) | 215 |
| **#9** | #7 OR #8 | 609119 |
| **#10** | #3 AND #6 AND #9 | 177 |

Table S1.4 Cochrane Library 205

| **Search Number** | **Query** | **Results** |
| --- | --- | --- |
| **#1** | MeSH descriptor: [Myocardial Ischemia] explode all trees | 39,246 |
| **#2** | MeSH descriptor: [Coronary Artery Disease] explode all trees | 9,734 |
| **#3** | MeSH descriptor: [Acute Coronary Syndrome] explode all trees | 3,177 |
| **#4** | MeSH descriptor: [Myocardial Infarction] explode all trees | 15,836 |
| **#5** | MeSH descriptor: [Angina Pectoris] explode all trees | 5,596 |
| **#6** | MeSH descriptor: [Percutaneous Coronary Intervention] explode all trees | 9,024 |
| **#7** | MeSH descriptor: [Coronary Artery Bypass] explode all trees | 6,813 |
| **#8** | ("Myocardial Ischemias" OR "Heart Diseases, Ischemic" OR "Ischemias, Myocardial" OR "Diseases, Ischemic Heart" OR "Disease, Ischemic Heart" OR "Ischemia, Myocardial" OR "Ischemic Heart Disease" OR "Heart Disease, Ischemic" OR "Ischemic Heart Diseases" OR "Arteriosclerosis, Coronary" OR "Arterioscleroses, Coronary" OR "Coronary Arteriosclerosis" OR "Coronary Atheroscleroses" OR "Coronary Atherosclerosis" OR "Atherosclerosis, Coronary" OR "Coronary Arterioscleroses" OR "Atheroscleroses, Coronary" OR "Left Main Disease" OR "Left Main Coronary Disease" OR "Left Main Diseases" OR "Left Main Coronary Artery Disease" OR "Artery Diseases, Coronary" OR "Artery Disease, Coronary" OR "Coronary Artery Diseases" OR "Syndrome, Acute Coronary" OR "Coronary Syndromes, Acute" OR "Coronary Syndrome, Acute" OR "Syndromes, Acute Coronary" OR "Acute Coronary Syndromes" OR "Infarcts, Myocardial" OR "Heart Attacks" OR "Stroke, Cardiovascular" OR "Infarct, Myocardial" OR "Cardiovascular Strokes" OR "Infarction, Myocardial" OR "Myocardial Infarct" OR "Strokes, Cardiovascular" OR "Infarctions, Myocardial" OR "Myocardial Infarcts" OR "Myocardial Infarctions" OR "Heart Attack" OR "Cardiovascular Stroke" OR "Angor Pectoris" OR "Stenocardias" OR "Stenocardia" OR "Coronary Revascularization, Percutaneous" OR "Revascularization, Percutaneous Coronary" OR "Percutaneous Coronary Revascularizations" OR "Interventions, Percutaneous Coronary" OR "Percutaneous Coronary Revascularization" OR "Coronary Intervention, Percutaneous" OR "Coronary Interventions, Percutaneous" OR "Revascularizations, Percutaneous Coronary" OR "Intervention, Percutaneous Coronary" OR "Coronary Revascularizations, Percutaneous" OR "Percutaneous Coronary Interventions" OR "Bypasses, Aortocoronary" OR "Bypass, Coronary Artery" OR "Aortocoronary Bypass" OR "Artery Bypass, Coronary" OR "Bypass, Aortocoronary" OR "Coronary Artery Bypass Grafting" OR "Coronary Artery Bypasses" OR "Bypasses, Coronary Artery" OR "Artery Bypasses, Coronary" OR "Coronary Artery Bypass Surgery" OR "Bypass Surgery, Coronary Artery" OR "Aortocoronary Bypasses"):ti,ab,kw (Word variations have been searched) | 78,898 |
| **#9** | #1 OR #2 OR #3 OR #4 OR #5 OR #6 OR #7 OR #8 | 84,236 |
| **#10** | MeSH descriptor: [Telemedicine] explode all trees | 5,984 |
| **#11** | ("Medicine, Virtual" OR "Tele-Referrals" OR "Tele Referral" OR "Virtual Medicine" OR "Tele-Referral" OR "Tele-Intensive Care" OR "Tele ICU" OR "Tele Intensive Care" OR "Tele-ICU" OR "Telecare" OR "Tele-Care" OR "Tele Care" OR "Health, Mobile" OR "mHealth" OR "Mobile Health" OR "Telehealth" OR "eHealth"):ti,ab,kw (Word variations have been searched) | 11,727 |
| **#12** | #10 OR #11 | 2,610 |
| **#13** | #9 AND #12 | 205 |

Table S1.5 Medline 199

| **Search Number** | **Query** | **Results** |
| --- | --- | --- |
| **#1** | exp Myocardial Ischemia/ | 496,875 |
| **#2** | exp Coronary Artery Disease/ | 84,571 |
| **#3** | exp Acute Coronary Syndrome/ | 22,541 |
| **#4** | exp Myocardial Infarction/ | 204,646 |
| **#5** | exp Angina Pectoris/ | 45,410 |
| **#6** | exp Percutaneous Coronary Intervention/ | 70,898 |
| **#7** | exp Coronary Artery Bypass/ | 58,830 |
| **#8** | ("myocardial ischemia" or "heart disease, ischemic" or "disease, ischemic heart" or "diseases, ischemic heart" or "heart diseases, ischemic" or "ischemic heart diseases" or "ischemia, myocardial" or "ischemias, myocardial" or "myocardial ischemias" or "ischemic heart disease" or "coronary artery disease" or "artery disease, coronary" or "artery diseases, coronary" or "coronary artery diseases" or "coronary arteriosclerosis" or "arterioscleroses, coronary" or "coronary arterioscleroses" or "arteriosclerosis, coronary" or "atherosclerosis, coronary" or "atheroscleroses, coronary" or "coronary atheroscleroses" or "coronary atherosclerosis" or "left mainleft main coronary disease" or "left main disease" or "left main diseases" or "acute coronary syndrome" or "acute coronary syndromes" or "coronary syndrome, acute" or "coronary syndromes, acute" or "syndrome, acute coronary" or "syndromes, acute coronary" or "myocardial infarction" or "infarction, myocardial" or "infarctions, myocardial" or "myocardial infarctions" or "heart attack" or "heart attacks" or "myocardial infarct" or "infarct, myocardial" or "infarcts, myocardial" or "myocardial infarcts" or "cardiovascular stroke" or "cardiovascular strokes" or "stroke, cardiovascular" or "strokes, cardiovascular" or "angina pectoris" or "angor pectoris" or "stenocardia" or "stenocardias" or "percutaneous coronary intervention" or "coronary intervention, percutaneous" or "coronary interventions, percutaneous" or "intervention, percutaneous coronary" or "interventions, percutaneous coronary" or "percutaneous coronary interventions" or "percutaneous coronary revascularization" or "coronary revascularization, percutaneous" or "coronary revascularizations, percutaneous" or "percutaneous coronary revascularizations" or "revascularization, percutaneous coronary" or "revascularizations, percutaneous coronary" or "coronary artery bypass" or "artery bypass, coronary" or "artery bypasses, coronary" or "bypasses, coronary artery" or "coronary artery bypasses" or "coronary artery bypass grafting" or "coronary artery bypass surgery" or "aortocoronary bypass" or "aortocoronary bypasses" or "bypass, aortocoronary" or "bypasses, aortocoronary" or "bypass surgery, coronary artery" or "bypass, coronary artery").ab,ti. | 475,228 |
| **#9** | 1 or 2 or 3 or 4 or 5 or 6 or 7 or 8 | 692,164 |
| **#10** | exp Telemedicine/ | 56,535 |
| **#11** | ("telemedicine" or "virtual medicine" or "medicine, virtual" or "tele-referral" or "tele referral" or "tele-referrals" or "mobile health" or "health, mobile" or "mhealth" or "telehealth" or "ehealth" or "tele-intensive care" or "tele intensive care" or "tele-icu" or "tele icu" or "telecare" or "tele-care" or "tele care").ab,ti. | 56,449 |
| **#12** | 10 or 11 | 82,168 |
| **#13** | exp Randomized Controlled Trial/ | 657,489 |
| **#14** | randomized controlled trial.ab,ti. | 138,342 |
| **#15** | 13 or 14 | 709,729 |
| **#16** | 9 and 12 and 15 | 199 |

# Section S2 — Corrected Covered Area

We calculated the Corrected Covered Area (CCA) following Pieper et al l[1]. 10 to quantify the degree of citation overlap between the present review and prior systematic reviews. Of the nine prior reviews [2-10], Shi et al[7]. is an umbrella review and does not independently search for primary RCTs; it was excluded from the computation, yielding c = 8. The formula used was:

where r = 13 (primary studies in the present review), c = 8 (prior reviews compared), and N = 19 (total cumulative inclusions of the 13 primary studies across the 8 prior reviews).

This falls within the 6–10% moderate overlap range. Of the 13 included trials, six were not included in any prior review. The overlap matrix is shown below.

CCA = (19 − 13) / (13 × 8 − 13) = 6 / 91 = 6.6%

| Included study | Li[2] | Cruz[3] | Gao[4] | Gong[5] | Ram[6] | Xu[8] | Yang[9] | Yu[10] | Sum |
| --- | --- | --- | --- | --- | --- | --- | --- | --- | --- |
| Avila et al 2018 [31] | ✓ |  |  | ✓ |  |  |  | ✓ | 3 |
| Li et al 2026 [43] |  |  |  |  |  |  |  |  | 0 |
| Dehghani et al 2024 [32] |  |  |  |  |  |  |  |  | 0 |
| Duscha et al 2018 [33] | ✓ | ✓ |  |  |  |  |  | ✓ | 3 |
| Frederix et al 2015 [34] | ✓ |  |  |  |  |  | ✓ | ✓ | 3 |
| Fang et al 2019 [35] |  | ✓ |  | ✓ | ✓ |  |  |  | 3 |
| Maddison et al 2015 [36] | ✓ | ✓ |  |  |  |  |  |  | 2 |
| Nabutovsky et al 2024 [37] |  |  |  |  |  |  |  |  | 0 |
| Salvetti et al 2008 [38] |  |  |  |  |  |  |  |  | 0 |
| Song et al 2020 [39] | ✓ | ✓ |  | ✓ | ✓ |  |  |  | 4 |
| Wan et al 2025 [40] |  |  |  |  |  |  |  |  | 0 |
| Yao et al 2026 [41] |  |  |  |  |  |  |  |  | 0 |
| Zutz et al 2007 [42] |  |  |  |  | ✓ |  |  |  | 1 |

# Section S3 — Adverse Events and Adherence Reported in the Included Studies on Home-Based Cardiac Telerehabilitation

**Table S1. Summary of adverse events, compliance, and dropout rates included in the study**

| **Study (First author, Year)** | | **Country** | | **Sample size (Total / Int. / Ctrl.)** | **Intervention & Duration** | | **Adverse Events Reported** | **Adherence / Compliance** | **Dropouts / Withdrawal** | | **Notes** |
| --- | --- | --- | --- | --- | --- | --- | --- | --- | --- | --- | --- |
| **Avila et al., 2018 [31]** | | Belgium | | N = 90  HB: 30  CB: 30  CG: 30 | Home-based exercise + telemonitoring (weekly email/phone) vs. centre-based CR vs. usual care  Duration: 12 weeks | | **Explicitly reported:**  "No serious adverse events related to exercise occurred in any of the groups." | **Reported in detail:**  HB group: mean 2.5 sessions/week (range 12–60 over 12 wks); 164 min/week at avg 46.8% HRR; 76.7 min/week within prescribed zone  CB group: mean 2.0 sessions/week (range 4–36); 90 min/week at avg 61.2% HRR | Total: 6/90 (6.7%)  • HB: 2/30 (loss of interest)  • CB: 0/30  • CG: 4/30 (2 loss of interest; 2 new cardiac intervention/PCI) | | Heart rate monitors used in HB group only; CB group used palpation. Authors note inability to define exact minutes within prescribed training zone for CB. |
| **Dehghani et al., 2024 [32]** | | Iran | | Initially recruited: 155  Eligible/randomised: 103  Analysed: 80  Int.: 40  Ctrl.: 40 | Home-based cardiac telerehabilitation (HBCT): supervised exercise, walking, phone calls, pedometer vs. usual care (walking advice only)  Duration: 8 weeks | | **Explicitly reported:**  "we did not record any adverse events during physical exercises, indicating the safety and feasibility of the telerehabilitation method." | **Pre-specified adherence threshold:**  Patients attending <25% of rehabilitation sessions were excluded as non-adherent.  N = 58 excluded for lack of adherence (from initial 155 → 80 analysed).  Quantitative session-level adherence rates for the 80 retained patients not separately reported. | Reported as exclusions for non-adherence (<25% sessions): n = 58.  Specific dropout numbers within the analysed sample (n = 80) not separately reported. | | Conducted during COVID-19 pandemic. Single-blinded (statistician). Pedometer-based progressive walking program with stepwise increase from 3,000 to 8,250 steps/day. |
| **Duscha et al., 2018 [33]** | | USA | | Screened: 503  Randomised: 32  Analysed: 25  mHealth: 16  Usual care: 9  (3:1 randomisation) | mHealth post-CR: Fitbit activity tracker + step-based exercise prescription + weekly health coaching (Vida Health) vs. usual care  Duration: 12 weeks (post-CR completion) | | **Not explicitly reported as a category.**  Events leading to dropout described, but no formal adverse-event reporting:  1 patient: knee injury after falling on ice  1 patient: diagnosis of rare blood disease  1 patient: severe wound from fishing-hook accident  (none clearly attributable to the intervention) | **Medication adherence:**  Morisky Medication Adherence Questionnaire — high adherence at end of CR; no significant change at 12 weeks within or between groups.  **Exercise adherence:**  Step counts objectively monitored via Fitbit; mHealth group sustained ~9,414 steps/day at 12 weeks. Specific session-level adherence rate not reported. | Total dropouts: 5/32 (15.6%)  mHealth: 3 (knee injury; blood disease; lost to follow-up)  Usual care: 2 (fishing hook accident; lost to follow-up)  Plus 2 with unusable data (mHealth: 1 ICD reset; 1 inadequate effort on CPX) | | Pilot study. Partially funded by Vida Health, Inc. Two of the dropouts (knee injury, fishing-hook wound) were unrelated to study procedures. |
| **Fang et al., 2019 [35]** | | China | | N = 80 (recruited)  HBCTR: 40 → 33 analysed  UC: 40 → 34 analysed | HBCTR: belt-strap sensor + smartphone app + outdoor walking/jogging with real-time monitoring + biweekly home visits + weekly phone calls vs. usual care (CHD booklet + biweekly outpatient review)  Duration: 6 weeks | | **Not reported.**  No statement on adverse events, exercise-related complications, or safety outcomes provided in the manuscript. | **Not quantitatively reported.**  Intervention prescribed walking/jogging ≥3 times/week for 6 weeks with real-time physiological monitoring and weekly phone-call support.  Authors describe "good adherence" in the conclusion but provide no session-level or step-level adherence data. | UC group: 5/40 (12.5%)  1 excluded; 3 withdrew; 1 lost to follow-up  HBCTR group: 6/40 (15.0%)  1 excluded; 4 withdrew; 1 lost to follow-up  Reasons for withdrawal not specified. | | Authors acknowledge limitation: small sample, reliance on self-reported scales (FTND, CDS, SF-36). Failure to report adverse events and quantitative adherence is a notable gap. |
| **Frederix et al., 2015 [34]** | | Belgium | | Assessed: 1,247  Randomised: 80  Analysed: 66  Int.: 32  Ctrl.: 34 | Physical activity telemonitoring: 3D accelerometer (Yorbody) worn continuously + automated weekly email/SMS feedback + 10%/week step-count goal increase, in addition to conventional CR vs. conventional CR alone  Duration: 18 weeks | | **Not explicitly reported as a discrete adverse-event category.**  Re-hospitalisations within 125 days reported as a safety/efficacy proxy:  Int.: 4/32 (12.5%); 2 cardiovascular (angina)  Ctrl.: 9/34 (26.5%); 6 cardiovascular (angina, restenosis, syncope, drug side-effect)  Trend favouring intervention (P = 0.09). | **Adherence not quantitatively reported.**  All randomised patients in the intervention group were instructed to wear the motion sensor continuously (except sleep/bathing) and to upload weekly. Specific compliance rates for sensor wear or step-goal achievement not reported. | Total: 14/80 (17.5%)  Intervention: 8/40 (20.0%)  5 new pathology  2 loss of interest  1 ICT problem  Control: 6/40 (15.0%)  3 new pathology  3 loss of interest | | Authors did not separately report exercise-related adverse events. Re-hospitalisation analysis used as a clinical-event endpoint. Motion sensors provided free of charge by Yorbody. |
| Li et al., 2026 [43] | | China | | N = 68  Int.: 34  Ctrl.: 34 | Nurse-led, mHealth-based CR (HeartFIT) via WeChat: individualized exercise prescription + health education + goal-setting + remote nursing support vs. usual care  Duration: 12 weeks | | **Explicitly reported:** No serious adverse events occurred during the intervention period.  During baseline CPET: 1 participant experienced hypoglycemia; 1 developed oxygen desaturation (83%) with mild dyspnea at peak exercise; both were managed with rest, oxygen and energy supplementation, with complete resolution and successful retesting. | Adherence rate: 84.4% of prescribed sessions completed (among those who completed follow-up).  2 participants showed poor adherence (<80% of sessions), attributed to scheduling difficulties and waning motivation; excluded from per-protocol analysis. | Total: 6/68 (8.8%), retention rate 91.2%  Int.: 2 (1 lost contact; 1 withdrew)  Ctrl.: 4 (2 lost contact; 2 withdrew)  Additional 5 unable to attend final in-person CPET due to COVID-19 restrictions (2 Int.; 3 Ctrl.) | | Pediatric population (age 8-18 yrs). Per-protocol analysis (n=55) confirmed robustness. Study conducted during COVID-19 pandemic. |
| Maddison et al., 2015 [36] | | New Zealand | | N = 171  Int.: 85  Ctrl.: 86 | Mobile phone-delivered exercise CR (HEART): personalized automated text messages (118 over 24 wks) + secure website with video messages aimed at increasing exercise behaviour vs. usual care (community-based CR)  Duration: 24 weeks | | **Explicitly reported**: 22 participants reported 31 serious adverse events total.  15 were cardiac-related (angina/chest pain=4; stenosis=2; heart palpitations=1; shortness of breath=2; dizziness=1; pericarditis=3; ventricular tachycardia=2).  Remaining events: cancer diagnosis (n=3); injury (n=3); other illness (n=10).  Only 1 serious adverse event was related to study treatment: participant hospitalized following a cycling accident. | 82% of intervention participants read some or all HEART text messages.  57% viewed some or all website video messages.  Average website use: once every two weeks.  Specific session-level adherence rate not reported. | Int.: 10/85 lost to follow-up  (could not contact n=5; unable to complete assessment n=4; did not wish to return n=1)  Ctrl.: 8/86 lost to follow-up  (could not contact n=3; unable to complete assessment n=4; did not wish to return n=1)  Primary outcome data: Int. 75/85 (88%); Ctrl. 78/86 (91%) | | Adults (mean age 60 yrs). Single-blind RCT. Null effect for PVO2; significant effect for LTPA and walking. Post-hoc economic evaluation included. |
| Nabutovsky et al., 2024 [37] | | Israel | | N = 69 randomised  Int.: 45 (42 analysed)  Ctrl.: 24 (21 analysed) | Asynchronous HBCR (Datos Health platform): structured exercise monitored by smartwatch (Garmin/Polar) + remote multidisciplinary team follow-up + educational mobile app content + phone coaching vs. usual care (general exercise prescription + cardiologist consultations)  Duration: 6-month active HBCR + 6-month follow-up | | **Explicitly reported:** No hospitalizations or deaths related to the intervention or physical activity occurred during the study.  4 participants (2 Int., 2 Ctrl.) were hospitalized briefly (1-4 days) for reasons unrelated to the intervention. | Median aerobic exercise: 193.2 min/week (IQR 110.2-251.5) = 129% of 150 min/week goal.  Median time in target HR zone: 112 min/week (100% of weekly goal).  Median workout intensity: 71% of max HR during CPET (100% of goal).  Median aerobic workouts/week: 4.9; resistance training: 0.65/week (32.5% of 2/week goal).  Median daily steps: 9252 (116% of 8000/day goal). | Int.: 3/45 discontinued (lack of engagement / unwillingness to cooperate with care team)  Ctrl.: 3/24 discontinued (same reasons) | | Unique population: patients who actively declined CBCR. 62% moderate-to-high CV risk. Baseline VO2peak lower in Int. vs. Ctrl. (likely random difference due to small sample). |
| Salvetti et al., 2008 [38] | | Brazil | | N = 39  Int. (home): 19  Ctrl.: 20 | Home-based exercise training: 2 initial supervised classes + 3-month unsupervised walking (3x/week, 30 min, 60-80% peak HR) + biweekly telephone monitoring by physician vs. standard care (no active intervention)  Duration: 3 months | | **Explicitly reported**: No training-related cardiac events occurred in the home group.  No coronary events occurred in either group during the experimental period. | Average total adherence: 100% (per patient log books confirmed by phone call monitoring).  Home group mean sessions: 2.8 (SD 0.4) sessions/week.  Control group physical activity habits not quantified to avoid influencing their behaviour. | No dropouts reported.  All 39 randomised participants completed 3-month outcome assessment (n=19 home; n=20 control). | | Low-risk patients only (NYHA I-II, EF >50%, no HF). Two supervised classes provided before unsupervised home phase. Biweekly telephone monitoring by physician. Average cost per patient: US$502.71 (BHCMP). |
| Song et al., 2020 [39] | | China | | N = 96 (analysed)  Int.: 48  Ctrl.: 48 | Smartphone-based telemonitored exercise rehabilitation (MEMRS-CRS + HR belt): exercise prescription based on CPET (walking at HR@AT +/- 5 bpm, 3-5x/week, 30 min) + weekly feedback via WeChat/telephone vs. routine clinical follow-up  Duration: 6 months | | **Explicitly reported:** No serious complications or adverse events occurred during follow-up. | Exercise frequency: 5.1 +/- 0.6 times/week in intervention group.  Exercise duration: 31.4 +/- 4.5 min/session.  Time within target HR zone: 94.2 +/- 6.4% of total exercise time.  91.7% (44/48) of intervention participants satisfied with content and frequency of feedback.  Proportion with exercise habits at 6 months: Int. 93.8% vs. Ctrl. 77.1% (P=0.021). | Total: 10/106 enrolled (9.4%); 5 per group.  Int. dropouts: 1 unable to use monitoring software; 1 withdrew for work reasons; 3 failed to continuously wear monitoring device.  Ctrl. dropouts: 2 withdrew for work/family reasons; 3 refused hospital follow-up at 6 months. | | Stable CHD patients in China. Longer intervention period than most telemonitored studies (6 months). No explicit economic cost analysis. Telemonitoring-specific compliance: 91.7% satisfied with feedback. |
| **Wan et al., 2025 [40]** | | China | | N = 137 enrolled  Int.: 65  Ctrl.: 65  (130 completed) | WeChat-based brisk walking cardiac rehabilitation (CR) vs. usual care (personalised exercise plan, no telemonitoring)  Duration: 12 weeks | | **Adverse Events**  **Explicitly reported:** "No adverse cardiovascular events happened throughout the entire intervention."  **Safety protocol:** A thorough adverse event protocol was created to protect participants' safety. Heart rate was monitored via smartphone app; nurses issued alerts when heart rate approached maximum during exercise. | **Adherence / Compliance**  **Engagement:** Participants uploaded exercise training records (resting/exercise HR, BP) to WeChat group as required.  **Prescription adherence:** Wks 1–4: brisk walking 30 min/session, 3 days/week; Wks 5–12: 5 days/week. No quantitative session-level adherence rate reported.  **WeChat log-in/upload rate:** Not reported. | **Dropouts**  Total dropouts: 7/137 (5.1%)  Int.: 3 (reasons not specified)  Ctrl.: 4 (reasons not specified)  Retention: 130/137 (94.9%) | | Single-centre RCT; single-blind. All-Chinese population. Primary outcome: TSK-SV Heart kinesiophobia score. Secondary: Self-Efficacy for Exercise (SEE) and peak VO2. No long-term follow-up beyond 12 weeks. Multidisciplinary team (nurse, CR physician, therapist, specialist nurses, research assistants). |
| **Yao et al., 2026 [41]** | China | | N = 62  Int.: 31  Ctrl.: 31  (ITT analysis) | | | AI-assisted telerehabilitation ("Rehabilitation Exercise Prescription" app with 3D skeletal pose recognition, real-time feedback, personalised exercise prescriptions) + health education vs. conventional offline exercise guidance + health education  Duration: 8 weeks | **Adverse Events**  **Explicitly reported:** "Throughout the 8-week intervention period, no serious adverse events related to the AI-assisted telerehabilitation intervention occurred."  **Monitoring:** Weekly online consultations or telephone follow-ups conducted. Exercise intensity adjusted based on individual feedback. No wearable HR monitoring used (noted as a limitation). | **Adherence / Compliance**  **Prescription frequency:** Exercise pushed 5×/week, required ≥3×/week, 30–50 min/session.  **Session-level adherence rate:** Not formally reported as a percentage.  **Indirect indicator:** Total exercise time significantly greater in intervention vs. control (adjusted mean difference 98.24 s, 95% CI 49.39–147.08; P = .001).  **App engagement:** Automated reminders and post-session reports generated; adherence facilitated by closed-loop monitoring–feedback–prompting system. Quantitative log-in or session completion rate not reported. | | **Dropouts**  Total dropouts: 7/62 (11.3%)  Int.: 3 (2 sudden illness; 1 personal reasons)  Ctrl.: 4 (1 physical reasons; 3 personal reasons)  • ITT principle applied; missing data handled by multiple imputation. | Multicenter RCT (2 hospitals, Guangdong, China). Open-label design. Primary outcome: 6-minute walk distance (6MWD). CPET performed in subset only (n = 24/62). Convenience sampling. No financial compensation for participants. AI app provided by RaphAI Health Technologies. Limitations: small sample, short follow-up, no objective HR monitoring during exercise. |
| **Zutz et al., 2007 [42]** | | Canada | | N = 15  vCRP: 8  Ctrl.: 7  (+16 historical controls) | Internet-based virtual CRP (vCRP): online intake forms + one-on-one chat sessions (nurse, dietitian, exercise specialist) + downloadable HR monitoring + weekly education + data monitoring (BP, weight, glucose) vs. observational control (wait-listed, no contact)  Duration: 12 weeks | | **Adverse Events**  **Explicitly reported:** "There were no adverse events reported" and "There were no changes in medications in any of the study participants."  **Safety design:** High-risk individuals excluded (uncontrolled diabetes, significant arrhythmia, ≥2 mm ST-depression). Exercise HR data uploaded to web server and reviewed by exercise specialist for target HR compliance. | **Adherence / Compliance**  **Log-in frequency:** Average 50 log-ins over 12 weeks = 4.2 times/week (range: 26–86 log-ins).  **Weekly task completion:** Average 66% of weekly tasks completed (range: 12.5%–100%); tasks included intake forms, HR upload, BP/weight data entry.  **Exercise outcomes (indirect):** Physical activity increased >6-fold vs. baseline in vCRP group (982 → 6018 kcal/week; P < 0.05), substantially greater than controls (1720 → 2498 kcal/week). | **Dropouts**  CRP group: 0/8 dropouts (all completed 12 weeks)  Control group: 2/7 lost to follow-up (could not be contacted; also did not attend hospital CRP)  Historical controls: all 16 included for comparison | | Pilot RCT; small sample limits hypothesis testing. Participants provided with laptop, HR monitor (Polar), BP monitor, and dial-up internet — not a sustainable implementation model. Historical controls (n=16) matched 2:1 (age, sex, disease) for comparison. User feedback unanimously positive. Primary technology complaints: server connection reliability (75%). Precursor to modern telerehabilitation studies. |

**Note: AE = Adverse Event; BP = Blood Pressure; CBCR = Centralized Cardiac Rehabilitation; CHD = Coronary Artery Disease; Ctrl. = Control Group; CPET = Cardiopulmonary Exercise Test; CR = Cardiac Rehabilitation; CRP = Cardiac Rehabilitation Program; EF = Ejection Fraction; HBCR / HBCTR = Home-based Cardiac (Remote) Rehabilitation; HR = Heart Rate; HRR = Heart Rate Reserve; ICD = Implantable Cardiac Defibrillator; ICT = Information and Communication Technology; Int. = Intervention Group; ITT = Intention-to-Treatment; LTPA = Leisure Time Physical Activity; mHealth = Mobile Health; N = Total Number; NYHA = New York Heart Association Classification; PCI = Percutaneous Coronary Intervention; PVO₂ / VO₂peak = Peak Oxygen Uptake; RCT = Randomized Controlled Trial; SD = Standard Deviation; vCRP = Virtual Cardiac Rehabilitation Program; WeChat = WeChat.**

# Section S4 — Grading of Recommendations, Assessment, Development, and Evaluations

**Author(s):** Zhicheng Zhu

**Question:** A comparison of peak oxygen uptake, systolic blood pressure, and diastolic blood pressure in cardiovascular disease patients between telemedicine and routine medical care.

**Setting:**

**Bibliography:**

| **Certainty assessment** | | | | | | | **№ of patients** | | **Effect** | | **Certainty** | **Importance** |
| --- | --- | --- | --- | --- | --- | --- | --- | --- | --- | --- | --- | --- |
| **№ of studies** | **Study design** | **Risk of bias** | **Inconsistency** | **Indirectness** | **Imprecision** | **Other considerations** | **Telerehabilitation with Exercise as the Core Component** | **usual care OR without structured exercise training** | **Relative (95% CI)** | **Absolute (95% CI)** |  |  |
| **Peak oxygen uptake** | | | | | | | | | | | | |
| 10 | randomised trials | not serious | serious^a^ | serious^b^ | serious^c^ | publication bias strongly suspected | 387 | 362 | - | MD **2.58 higher higher** (1.28 higher to 3.88 higher) | ⨁◯◯◯ Very low^a,b,c^ |  |
| **Systolic blood pressure** | | | | | | | | | | | | |
| 5 | randomised trials | not serious | serious^d^ | serious^e^ | serious^f^ | publication bias strongly suspected | 148 | 147 | - | MD **1.8 lower more** (6.13 fewer to 2.53 more) | ⨁◯◯◯ Very low^d,e,f^ |  |
| **Diastolic blood pressure** | | | | | | | | | | | | |
| 5 | randomised trials | not serious | not serious | very serious^g^ | serious^h^ | publication bias strongly suspected | 148 | 147 | - | MD **2 lower higher** (4.34 lower to 0.34 higher) | ⨁◯◯◯ Very low^g,h^ |  |

**CI:** confidence interval; **MD:** mean difference

#### Explanations

a. An I² of 87.3% indicates high heterogeneity, and τ² of 2.5114 suggests substantial real differences among the studies, which cannot be explained by sampling error. Most importantly, the direction of the effects is inconsistent: the Nabutovsky et al [37] (−0.30) and Maddison et al [36] (−0.10) studies showed that remote rehabilitation was ineffective or even slightly disadvantageous, contrary to the positive results of the other eight studies, and cannot be explained solely by random fluctuations. The effect size ranged from −0.30 to +5.00, exhibiting extreme variability.

b. Indirectness rated down by one level. Population: included trials enrolled a heterogeneous spectrum of cardiovascular disease subtypes (coronary artery disease, heart failure, post-PCI, hypertension) together with one pediatric congenital heart disease cohort, limiting directness to any single adult CVD population of interest. Intervention: 8 of 13 trials embedded exercise within multicomponent packages incorporating health education, dietary counselling, and behavioural support; only 2 trials delivered exercise as a sole intervention. Delivery platforms (smartphone apps, wearables, SMS, web, telephone), supervision models, and exercise prescriptions varied substantially, so the pooled effect cannot be unambiguously attributed to the exercise stimulus alone. Outcome: VO₂peak is a surrogate endpoint; although strongly and consistently associated with cardiovascular mortality, no trial reported direct patient-important outcomes such as mortality, myocardial infarction, or hospitalisation

c. The prediction interval {−1.38 - 6.54} spans the null threshold (0) and the MCID threshold (1.0), suggesting that the intervention may be meaningless or even harmful in certain clinical contexts. Although the lower limit of the 95% CI (1.16) superficially exceeds the MCID, in the context of high heterogeneity (I²=87.3%), this CI underestimates the true uncertainty—the prediction interval is the correct indicator of "the range of effects that may be observed in a similar future study," and its width (7.77 units) reveals the high degree of uncertainty of the outcome.

d. Only one of the five included studies — Yao et al [41] (2026) — specifically enrolled patients with hypertension and designated blood pressure reduction as a primary treatment objective. The remaining four studies recruited patients with coronary artery disease or ischaemic heart disease, in whom blood pressure was a secondary, incidentally measured outcome rather than a therapeutic target. This fundamental population mismatch limits the applicability of the pooled estimate to any well-defined target population. Additionally, the included interventions ranged from brief text-message programmes (Avila, 2018) to AI-guided exercise prescription (Yao, 2026), representing substantially different mechanistic pathways to any potential

e. The lower bound of the 95% confidence interval corresponds to a DBP reduction of only 0.27 mmHg — a magnitude that falls far below any accepted threshold for clinical importance (conservative MCID ≥2 mmHg; strict MCID ≥5 mmHg). Although the pooled point estimate of −2.76 mmHg surpasses the conservative MCID, the near-crossing of the null value renders the conclusion highly unstable. A small change in any single study — or the addition of one adequately powered null-result trial — would shift the inference across the line of no effect. The total analysed sample of approximately 292 participants is insufficient to establish a reliable estimate for a continuous cardiovascular outcome of this nature. The fixed-effects model, while appropriate given the I² value, may additionally underestimate true uncertainty by assuming a common underlying effect across populations with divergent baseline DBP levels (range: 116 to 184 mmHg across studies).

f. Statistical heterogeneity across the five included studies is negligible. I² = 3.44% indicates that virtually all observed variance in effect estimates is attributable to within-study sampling error rather than true between-study differences, and the Q-test does not approach statistical significance (Q (4) = 4.14, p = 0.39). Four of five studies report point estimates favouring a reduction in diastolic blood pressure, and the single positive estimate from Fang et al (2019 [35]; +1.80 mmHg) is small and fully compatible with random variation. The fixed-effects inverse-variance model is the analytically appropriate choice given this level of consistency. On statistical grounds, no downgrade for inconsistency is warranted.

g. h. A meaningful degree of population indirectness is present. Only Yao et al [41] (2026) specifically enrolled patients with a diagnosis of hypertension and designated blood pressure control as a primary treatment objective; the remaining four studies recruited patients with established coronary artery disease or ischaemic heart disease, populations in which diastolic blood pressure was a secondary, incidentally monitored endpoint rather than the target of the intervention. This mismatch limits the direct applicability of the pooled estimate to any coherent target population — particularly to the hypertension management setting where clinicians are most likely to draw on this evidence. The included interventions also varied substantially in delivery mode, ranging from passive text-message reminders (Avila, 2018) to AI-guided, real-time motion-capture exercise programmes (Yao, 2026), introducing further uncertainty about which intervention components drive the observed signal and whether results a

h. Although the 95% CI did not cross zero, the 95% prediction interval spanned from –1.38 to 6.54 mL/kg/min, indicating that in some future clinical settings the true effect could be null or slightly negative.

# References

31. Avila A, Claes J, Goetschalckx K, et al. Home-based rehabilitation with telemonitoring guidance for patients with coronary artery disease (short-term results of the TRiCH study): randomized controlled trial. J Med Internet Res. Jun 22, 2018;20(6):e225. [doi: 10.2196/jmir.9943] [Medline: 29934286]

32. Dehghani M, Cheraghi M, Shakarami A, Dehghani M, Namdari M. Effects of cardiac telerehabilitation during COVID-19 on cardiac hemodynamic and functional responses and quality of life: a randomized controlled trial. Ann Med Surg (Lond). Nov 2024;86(11):6537-6545. [doi: 10.1097/MS9.0000000000002235] [Medline: 39525786]

33. Duscha BD, Piner LW, Patel MP, et al. Effects of a 12-week mHealth program on peak VO2 and physical activity patterns after completing cardiac rehabilitation: a randomized controlled trial. Am Heart J. May 2018;199:105-114. [doi: 10.1016/j.ahj.2018.02.001] [Medline: 29754647]

35. Fang J, Huang B, Xu D, Li J, Au WW. Innovative application of a home-based and remote sensing cardiac rehabilitation protocol in Chinese patients after percutaneous coronary intervention. Telemed J E Health. Apr 2019;25(4):288-293. [doi: 10.1089/tmj.2018.0064] [Medline: 30192210]

34. Frederix I, Driessche NV, Hansen D, et al. Increasing the medium-term clinical benefits of hospital-based cardiac rehabilitation by physical activity telemonitoring in coronary artery disease patients. Eur J Prev Cardiolog. Feb 2015;22(2):150-158. [doi: 10.1177/2047487313514018]

43. Li Y, Zhou Y, Zhang Y, et al. Effectiveness of a nurse-led, mHealth-based cardiac rehabilitation program on cardiorespiratory fitness and physical activity in youth with congenital heart disease: a randomized controlled trial. Int J Nurs Stud. May 2026;177:105370. [doi: 10.1016/j.ijnurstu.2026.105370] [Medline: 41690191]

36. Maddison R, Pfaeffli L, Whittaker R, et al. A mobile phone intervention increases physical activity in people with cardiovascular disease: results from the HEART randomized controlled trial. Eur J Prev Cardiol. Jun 2015;22(6):701-709. [doi: 10.1177/2047487314535076] [Medline: 24817694]

37. Nabutovsky I, Breitner D, Heller A, et al. Home-based cardiac rehabilitation among patients unwilling to participate in hospital-based programs. J Cardiopulm Rehabil Prev. Jan 1, 2024;44(1):33-39. [doi: 10.1097/HCR.0000000000000796] [Medline: 37220026]

38. Salvetti XM, Oliveira JA, Servantes DM, Vincenzo de Paola AA. How much do the benefits cost? Effects of a homebased training programme on cardiovascular fitness, quality of life, programme cost and adherence for patients with coronary disease. Clin Rehabil. 2008;22(10-11):987-996. [doi: 10.1177/0269215508093331] [Medline: 18955430]

39. Song Y, Ren C, Liu P, Tao L, Zhao W, Gao W. Effect of smartphone-based telemonitored exercise rehabilitation among patients with coronary heart disease. J Cardiovasc Transl Res. Aug 2020;13(4):659-667. [doi: 10.1007/s12265-019- 09938-6] [Medline: 31820334]

40. Wan BY, Zhou C, Sun X, et al. The effect of WeChat-based brisk walking on kinesiophobia in patients after percutaneous coronary intervention: a randomised controlled trial. Front Cardiovasc Med. 2025;12:1416356. [doi: 10. 3389/fcvm.2025.1416356] [Medline: 40364826]

41. Yao Q, Qiu B, He L, et al. Effects of artificial intelligence recognition-based telerehabilitation on exercise capacity in patients with hypertension: randomized controlled trial. J Med Internet Res. Jan 13, 2026;28:e81400. [doi: 10.2196/ 81400] [Medline: 41529829]

42. Zutz A, Ignaszewski A, Bates J, Lear SA. Utilization of the internet to deliver cardiac rehabilitation at a distance: a pilot study. Telemed J E Health. Jun 2007;13(3):323-330. [doi: 10.1089/tmj.2006.0051] [Medline: 17603835]
