# Supplementary material for: Effects of Telerehabilitation With Exercise as the Core Component on Peak Oxygen Uptake and Blood Pressure in Patients With Cardiovascular Disease: Systematic Review and Meta-Analysis
Source: J Med Internet Res. 2026 Sep 21;28:e95923. doi: 10.2196/95923 (PMC13592150; doi:10.2196/95923)
Supplement: Supplementary file 2 [file jmir-v28-e95923-s002.docx]

# PRISMA 2020 Checklist

| **Section and Topic** | **Item #** | **Checklist item** | **Location where item is reported** |
| --- | --- | --- | --- |
| **TITLE** | | |  |
| Title | 1 | Identify the report as a systematic review. | Page 1, Line 1 |
| **ABSTRACT** | | |  |
| Abstract | 2 | See the PRISMA 2020 for Abstracts checklist. | Page 1, Lines 12–44 |
| **INTRODUCTION** | | |  |
| Rationale | 3 | Describe the rationale for the review in the context of existing knowledge. | Page 3, Lines 49–109 |
| Objectives | 4 | Provide an explicit statement of the objective(s) or question(s) the review addresses. | Page 4, Lines 110–151 |
| **METHODS** | | |  |
| Eligibility criteria | 5 | Specify the inclusion and exclusion criteria for the review and how studies were grouped for the syntheses. | Page 6-7, Lines 186–220 |
| Information sources | 6 | Specify all databases, registers, websites, organisations, reference lists and other sources searched or consulted to identify studies. Specify the date when each source was last searched or consulted. | Page 7, Lines 230–258 |
| Search strategy | 7 | Present the full search strategies for all databases, registers and websites, including any filters and limits used. | Page 8, Lines 260–266; Supplementary 1 |
| Selection process | 8 | Specify the methods used to decide whether a study met the inclusion criteria of the review, including how many reviewers screened each record and each report retrieved, whether they worked independently, and if applicable, details of automation tools used in the process. | Page 8, Lines 260–266 |
| Data collection process | 9 | Specify the methods used to collect data from reports, including how many reviewers collected data from each report, whether they worked independently, any processes for obtaining or confirming data from study investigators, and if applicable, details of automation tools used in the process. | Page 8, Lines 268–282 |
| Data items | 10a | List and define all outcomes for which data were sought. Specify whether all results that were compatible with each outcome domain in each study were sought (e.g. for all measures, time points, analyses), and if not, the methods used to decide which results to collect. | Page 8-9, Lines 284–288 |
|  | 10b | List and define all other variables for which data were sought (e.g. participant and intervention characteristics, funding sources). Describe any assumptions made about any missing or unclear information. | Page 9, Lines 289–293 |
| Study risk of bias assessment | 11 | Specify the methods used to assess risk of bias in the included studies, including details of the tool(s) used, how many reviewers assessed each study and whether they worked independently, and if applicable, details of automation tools used in the process. | Page 9, Lines 295–302 |
| Effect measures | 12 | Specify for each outcome the effect measure(s) (e.g. risk ratio, mean difference) used in the synthesis or presentation of results. | Page 9, Lines304–305 |
| Synthesis methods | 13a | Describe the processes used to decide which studies were eligible for each synthesis (e.g. tabulating the study intervention characteristics and comparing against the planned groups for each synthesis (item #5)). | Page 9, Lines 307–324 |
|  | 13b | Describe any methods required to prepare the data for presentation or synthesis, such as handling of missing summary statistics, or data conversions. | Page 9, Lines 312–322 |
|  | 13c | Describe any methods used to tabulate or visually display results of individual studies and syntheses. | Page 9-10, Lines 323–325 |
|  | 13d | Describe any methods used to synthesize results and provide a rationale for the choice(s). If meta-analysis was performed, describe the model(s), method(s) to identify the presence and extent of statistical heterogeneity, and software package(s) used. | Page 10, Lines 326-339 |
|  | 13e | Describe any methods used to explore possible causes of heterogeneity among study results (e.g. subgroup analysis, meta-regression). | Page 10, Lines 340–363 |
|  | 13f | Describe any sensitivity analyses conducted to assess robustness of the synthesized results. | Page 10, Line 364–365 |
| Reporting bias assessment | 14 | Describe any methods used to assess risk of bias due to missing results in a synthesis (arising from reporting biases). | Page 11, Lines 364–368 |
| Certainty assessment | 15 | Describe any methods used to assess certainty (or confidence) in the body of evidence for an outcome. | Page 11, Lines 370–373 |
| **RESULTS** | | |  |
| Study selection | 16a | Describe the results of the search and selection process, from the number of records identified in the search to the number of studies included in the review, ideally using a flow diagram. | Page 11, Lines 374–396; Figure 1 |
|  | 16b | Cite studies that might appear to meet the inclusion criteria, but which were excluded, and explain why they were excluded. | Page 11, Lines 394–402 |
| Study characteristics | 17 | Cite each included study and present its characteristics. | Page 12, Lines 409– 451; Table 1 |
| Risk of bias in studies | 18 | Present assessments of risk of bias for each included study. | Page 15, Lines 467–502; Figures 2–3 |
| Results of individual studies | 19 | For all outcomes, present, for each study: (a) summary statistics for each group (where appropriate) and (b) an effect estimate and its precision (e.g. confidence/credible interval), ideally using structured tables or plots. | Page 17, Lines 517–520; Figures 4–6 |
| Results of syntheses | 20a | For each synthesis, briefly summarise the characteristics and risk of bias among contributing studies. | Page 17, Lines 523–530, Page 19, Lines 553–557; Page 19-20, Lines 577–581; |
|  | 20b | Present results of all statistical syntheses conducted. If meta-analysis was done, present for each the summary estimate and its precision (e.g. confidence/credible interval) and measures of statistical heterogeneity. If comparing groups, describe the direction of the effect. | Page 17, Lines 531–542, Lines 537; Page 19, Lines 558–565; Page 20, Lines 582–589;Figures 4–6 |
|  | 20c | Present results of all investigations of possible causes of heterogeneity among study results. | Page 20-21, Lines 601–613; Table 2 |
|  | 20d | Present results of all sensitivity analyses conducted to assess the robustness of the synthesized results. | Page 24, Lines 703–721; Figure 8 |
| Reporting biases | 21 | Present assessments of risk of bias due to missing results (arising from reporting biases) for each synthesis assessed. | Page 23, Lines 685–695; Figure 7 |
| Certainty of evidence | 22 | Present assessments of certainty (or confidence) in the body of evidence for each outcome assessed. | Page 25, Lines 740–751; Supplementary 2 |
| **DISCUSSION** | | |  |
| Discussion | 23a | Provide a general interpretation of the results in the context of other evidence. | Page 26, Lines 757–791 |
|  | 23b | Discuss any limitations of the evidence included in the review. | Page 28- ,30 Lines 838–941 |
|  | 23c | Discuss any limitations of the review processes used. | Page 28- ,30 Lines 838–941 |
|  | 23d | Discuss implications of the results for practice, policy, and future research. | Page 31, Lines 942–962 |
| **OTHER INFORMATION** | | |  |
| Registration and protocol | 24a | Provide registration information for the review, including register name and registration number, or state that the review was not registered. | Page 1, Line 17; Page 4, Line 144 |
|  | 24b | Indicate where the review protocol can be accessed, or state that a protocol was not prepared. | Page 4, Line 144 |
|  | 24c | Describe and explain any amendments to information provided at registration or in the protocol. | Page 4, Lines 144–171 |
| Support | 25 | Describe sources of financial or non-financial support for the review, and the role of the funders or sponsors in the review. | Page 30, Lines 921–922 |
| Competing interests | 26 | Declare any competing interests of review authors. | Page 30, Lines 934 |
| Availability of data, code and other materials | 27 | Report which of the following are publicly available and where they can be found: template data collection forms; data extracted from included studies; data used for all analyses; analytic code; any other materials used in the review. | Page 30, Lines 936–937 |

# PRISMA-S Checklist

| **Section/topic** | **#** | **Checklist item** | **Location(s) Reported** |
| --- | --- | --- | --- |
| **INFORMATION SOURCES AND METHODS** | | | |
| Database name | 1 | Name each individual database searched, stating the platform for each. | Page 7, Lines 232–233 |
| Multi-database searching | 2 | If databases were searched simultaneously on a single platform, state the name of the platform, listing all of the databases searched. | N/A – databases were searched individually on separate platforms |
| Study registries | 3 | List any study registries searched. | Page 7, Lines 242–243 |
| Online resources and browsing | 4 | Describe any online or print source purposefully searched or browsed (e.g., tables of contents, print conference proceedings, web sites), and how this was done. | Page 7, Lines 244–248 (backward and forward citation search) |
| Citation searching | 5 | Indicate whether cited references or citing references were examined, and describe any methods used for locating cited/citing references (e.g., browsing reference lists, using a citation index, setting up email alerts for references citing included studies). | Page 7, Lines 242–243 |
| Contacts | 6 | Indicate whether additional studies or data were sought by contacting authors, experts, manufacturers, or others. | Not done; acknowledged as deviation from protocol (Page 7, Lines 249–255) |
| Other methods | 7 | Describe any additional information sources or search methods used. | None beyond those described |
| **SEARCH STRATEGIES** | | | |
| Full search strategies | 8 | Include the search strategies for each database and information source, copied and pasted exactly as run. | Supplementary 1 (Page 6, Line 220) |
| Limits and restrictions | 9 | Specify that no limits were used, or describe any limits or restrictions applied to a search (e.g., date or time period, language, study design) and provide justification for their use. | Page 7, Lines 235–240 (no language restriction imposed; searched from inception to March 20, 2026) |
| Search filters | 10 | Indicate whether published search filters were used (as originally designed or modified), and if so, cite the filter(s) used. | No published search filters were used |
| Prior work | 11 | Indicate when search strategies from other literature reviews were adapted or reused for a substantive part or all of the search, citing the previous review(s). | Not applicable; search strategy was developed de novo |
| Updates | 12 | Report the methods used to update the search(es) (e.g., rerunning searches, email alerts). | Not applicable; no search updates were performed |
| Dates of searches | 13 | For each search strategy, provide the date when the last search occurred. | Page 7, Line 235 (March 20, 2026) |
| **PEER REVIEW** | | | |
| Peer review | 14 | Describe any search peer review process. | No formal search peer review was conducted; acknowledged as limitation (Page 7-8, Lines 249–255) |
| **MANAGING RECORDS** | | | |
| Total Records | 15 | Document the total number of records identified from each database and other information sources. | Page 11, Lines 381–388; Figure 1 (1,303 records identified; 436 duplicates removed) |
| Deduplication | 16 | Describe the processes and any software used to deduplicate records from multiple database searches and other information sources. | Page 11, Line 381;(EndNote 25 used for deduplication) |

# PRISMA 2020 for Abstracts Checklist

| **Section and Topic** | **Item #** | **Checklist item** | **Reported (Yes/No)** |
| --- | --- | --- | --- |
| **TITLE** | | |  |
| Title | 1 | Identify the report as a systematic review. | **Yes** |
| **BACKGROUND** | | |  |
| Objectives | 2 | Provide an explicit statement of the main objective(s) or question(s) the review addresses. | **Yes** |
| **METHODS** | | |  |
| Eligibility criteria | 3 | Specify the inclusion and exclusion criteria for the review. | **No** |
| Information sources | 4 | Specify the information sources (e.g. databases, registers) used to identify studies and the date when each was last searched. | **Yes** |
| Risk of bias | 5 | Specify the methods used to assess risk of bias in the included studies. | **Yes** |
| Synthesis of results | 6 | Specify the methods used to present and synthesise results. | **Yes** |
| **RESULTS** | | |  |
| Included studies | 7 | Give the total number of included studies and participants and summarise relevant characteristics of studies. | **No** |
| Synthesis of results | 8 | Present results for main outcomes, preferably indicating the number of included studies and participants for each. If meta-analysis was done, report the summary estimate and confidence/credible interval. If comparing groups, indicate the direction of the effect (i.e. which group is favoured). | **Yes** |
| **DISCUSSION** | | |  |
| Limitations of evidence | 9 | Provide a brief summary of the limitations of the evidence included in the review (e.g. study risk of bias, inconsistency and imprecision). | **Yes** |
| Interpretation | 10 | Provide a general interpretation of the results and important implications. | **Yes** |
| **OTHER** | | |  |
| Funding | 11 | Specify the primary source of funding for the review. | **No** |
| Registration | 12 | Provide the register name and registration number. | **Yes** |
